# Unveiling the nanoworld of antimicrobial resistance: integrating nature and nanotechnology

**DOI:** 10.3389/fmicb.2024.1391345

**Published:** 2025-01-09

**Authors:** Devesh Sharma, Sakshi Gautam, Sakshi Singh, Nalini Srivastava, Abdul Mabood Khan, Deepa Bisht

**Affiliations:** ^1^Department of Biochemistry, ICMR-National JALMA Institute for Leprosy and Other Mycobacterial Diseases, Agra, India; ^2^School of Studies in Biochemistry, Jiwaji University, Gwalior, India; ^3^Division of Clinical Trials and Implementation Research, ICMR-National JALMA Institute for Leprosy and Other Mycobacterial Diseases, Agra, India

**Keywords:** microbial drug resistance, antimicrobial stewardship, nanoparticle drug delivery system, drug carriers, nanoparticles, synergisms, herbal compound, metabolites

## Abstract

A significant global health crisis is predicted to emerge due to antimicrobial resistance by 2050, with an estimated 10 million deaths annually. Increasing antibiotic resistance necessitates continuous therapeutic innovation as conventional antibiotic treatments become increasingly ineffective. The naturally occurring antibacterial, antifungal, and antiviral compounds offer a viable alternative to synthetic antibiotics. This review presents bacterial resistance mechanisms, nanocarriers for drug delivery, and plant-based compounds for nanoformulations, particularly nanoantibiotics (nAbts). Green synthesis of nanoparticles has emerged as a revolutionary approach, as it enhances the effectiveness, specificity, and transport of encapsulated antimicrobials. In addition to minimizing systemic side effects, these nanocarriers can maximize therapeutic impact by delivering the antimicrobials directly to the infection site. Furthermore, combining two or more antibiotics within these nanoparticles often exhibits synergistic effects, enhancing the effectiveness against drug-resistant bacteria. Antimicrobial agents are routinely obtained from secondary metabolites of plants, including essential oils, phenols, polyphenols, alkaloids, and others. Integrating plant-based antibacterial agents and conventional antibiotics, assisted by suitable nanocarriers for codelivery, is a potential solution for addressing bacterial resistance. In addition to increasing their effectiveness and boosting the immune system, this synergistic approach provides a safer and more effective method of tackling future bacterial infections.

## Graphical abstract

Targeted drug delivery approach through nanoantibiotics against drug resistant pathogens.
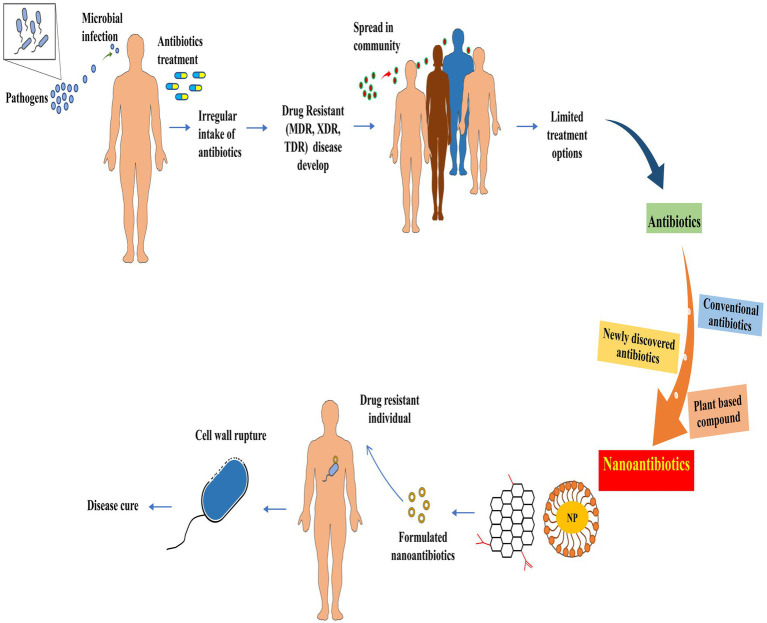

## 1 Introduction

Antimicrobial resistance (AMR) poses a severe threat to global health. The World Health Organization (WHO) lists AMR as one of the top 10 global health threats (World Health Organization, 2022; Michael et al., 2014). A study published in 2019 revealed that nearly 457,000 deaths were attributed to resistance caused by seven primary pathogens. These include *Escherichia coli*, *Staphylococcus aureus*, *Klebsiella pneumoniae*, *Pseudomonas aeruginosa*, *Enterococcus faecium*, *Streptococcus pneumoniae*, and *Acinetobacter baumannii*, listed in order of decreasing mortality (Antimicrobial Resistance Collaborators, 2022). Carbapenem resistance in *K. pneumoniae* is crucial to guiding antimicrobial selection, given how serious the threat is to public health (Karampatakis et al., 2023). The pathogenicity of carbapenem-resistant *K. pneumoniae* infections is enhanced by adhesive fimbriae, lipopolysaccharides, capsules, and siderophores. The formation of biofilms in patients with chronic or recurrent infectious diseases reflects bacterial resistance to antimicrobial drugs. Treatment strategies for this disease include traditional options (colistin and tigecycline) as well as newer alternatives (plazomicin and ceftolozane-tazobactam). Another major AMR concern is with microbes that cause urinary tract infections (UTIs) (Algammal et al., 2023). It is the second most prevalent infectious disease, caused by various gram-negative and gram-positive bacteria in all demographics. Notably, among the gram-negative bacteria, *E. coli* is a significant contributor to UTIs (Issakhanian and Behzadi, 2019).

With frequent infections and limited treatment options, AMR enforces alternate medication trials, prolonged hospital stays, and increased treatment costs. AMR arises from the overuse and misuse of antimicrobial drugs, inappropriate prescriptions, and insufficient knowledge of infection control. Repetitive antibiotic onslaughts apply genetic evolution pressure on the microbes, allowing them to acquire multidrug resistance (MDR) (Salam et al., 2023). The acquired genetic modification confers bacteria with either of the three main mechanisms: resistance, persistence, and antibiotic tolerance. Knowledge of antibiotic resistance’s exact mechanism and regulation strategies is vital for a suitable treatment strategy. Numerous studies were conducted to understand and control these mechanisms (Pang et al., 2018; Saha and Sarkar, 2021; Uddin et al., 2021). Traditional methods for diagnosing antibiotic resistance are slow, costly, and intricate.

Fortunately, nanotechnology emerged as a boon and a transformative opportunity, enhancing the speed and affordability of rapid point-of-care platforms. Nanoparticles (NPs) have dimensions typically in the 1 to 100 nm range (Boverhof et al., 2015; Teow et al., 2018). Their high surface area-to-volume ratio makes them versatile drug delivery vehicles (Ingle et al., 2013; Khan et al., 2019). NPs come in various categories: membrane-bound, metal-based, metal oxides, carbon, chitosan-based, and mesoporous structures. Additionally, formulations incorporate nanocomposites, nanosheets, nanomesh, hydrocarbons, and solid lipid NPs, each playing a role in enhancing antibiotic effectiveness, specificity, and delivery (Gabrielyan et al., 2020; Mamun et al., 2021; Parra-Ortiz et al., 2022). Recent advancements in NP vehicles have further increased their potency against drug-resistant bacteria (Wang et al., 2018; Brar et al., 2022; Binnebose et al., 2023). NP formulations can interact with cells through various mechanisms, such as adsorption, penetration, generation of reactive oxygen species, and interference with cellular processes (Barua and Mitragotri, 2014; Kim et al., 2015). The advantages include improved drug delivery, enhanced bioavailability, targeted therapy, prolonged drug release, improved drug stability, and minimized toxicity (Eleraky et al., 2020; Yeh et al., 2020). They have also been used as diagnostics and biosensing devices and as combination therapies against drugs in nanomaterials and nanoparticle formulations, biosensors, microfluidic devices, and so on. They have facilitated improved detection and treatment of antibiotic-resistant infections (Mubeen et al., 2021; Saxena et al., 2022). Nanoantibiotics (nAbts), a subfield of nanomedicine, are gaining attention due to their potential to revolutionize bacterial infection treatment (Edson and Kwon, 2016; Masri et al., 2019).

Nature has an abundance of compounds harboring antimicrobial properties. They are less toxic and more effective than synthetic drugs due to their evolution over time (Atanasov et al., 2021). The useful phytochemicals are sourced using ethnopharmacology and traditional medicine knowledge (Bonifácio et al., 2013; Nasim et al., 2022). Around one-third of popular pharmaceutical products are derived from natural sources, reflecting the growing demand for alternative healthcare solutions. Natural compounds are being studied for their medicinal potential in various health issues, including cancer and microbial diseases (Elkordy et al., 2021). Herb-based essential oils and secondary metabolites have antibacterial, antifungal, and antiviral properties, offering potential alternatives to traditional antibiotics (Joshi, 2016; Bhatwalkar et al., 2021; Magryś et al., 2021). Newman and Cragg (2016) reported that between 1981 and 2014, the FDA approved 1,562 pharmaceuticals, with 44% being unaltered natural products, 9.1% being botanical drugs, 21% being natural product derivatives, and 4% being synthetic drugs.

The shift from conventional to plant-based nanoformulations represents a noteworthy change in antimicrobial research and therapy. The abundant pharmacological possibilities in nature’s resources provide optimism for innovative therapies and improved healthcare outcomes. Green synthesis utilizes naturally sourced starting materials and low-energy processes as a sustainable alternative to conventional synthesis methods. This approach relies on a safer, cleaner, and more environmentally friendly nanomaterial manufacturing process (Huston et al., 2021; Chopra et al., 2022). Conjugation of these compounds with NPs shows more effectiveness than traditional antibiotics. They target multiple pathways in the body, reducing side effects such as liver or kidney damage, and are more biocompatible (Anand et al., 2022). This review aims to provide an extensive overview of innovative approaches, including nanocarriers and herbal compounds. It highlights the synergistic potential of combining multiple antibiotics within nanocarriers to maximize efficacy against drug-resistant pathogens. In addition, it emphasizes the importance of the green synthesis of NPs as a revolutionary method for enhancing antimicrobial effectiveness while minimizing the risk of systemic side effects. Unlike earlier reports, this review delves into the intricacies of antimicrobial resistance mechanisms while stressing the ethical utilization of natural resources and nanotechnology to address the challenge of drug resistance.

## 2 Antimicrobial resistance

The ability of pathogens to sustain and even counteract antibiotic activity poses the greatest challenge to the administration of drug therapy. The discovery of antibiotics and their extensive use have inadvertently facilitated the emergence of resistant pathogenic strains, which significantly challenge the current healthcare system and pose a serious environmental threat (Boucher et al., 2009; Huh and Kwon, 2011). Drug resistance is defined as the minimum inhibitory concentration (MIC) of antimicrobial agents exceeding the agent’s inhibitory effects, allowing the microorganism to persist and thrive (Andrews, 2001). The antibiotic resistance mechanism is divided into two sorts:

- Natural (or intrinsic): Cell wall or outer membrane thickening preventing antimicrobial entry (Nikolic and Mudgil, 2023); efflux pump (lipophilic and hydrophilic efflux pump) activation on the cell membrane (Nishino et al., 2021), inactivation of the drug (beta-lactamases hydrolyze the beta-lactam ring in penicillin and cephalosporins) (Bush and Bradford, 2016).
- Acquired: Comprises genetic material alterations (mutations, transformation, transposition, and conjugation) and biochemical mechanisms (secretion of alternative enzymes to degrade the concerned antibiotics; enzymatic modification such as methylation, adenylation, acetylation, etc., of target molecules; use of alternative pathways and quorum sensing; antibiotic sequestration) (Walsh, 2000; Munita and Arias, 2016; Kapoor et al., 2017; Peterson and Kaur, 2018).

### 2.1 Antibiotic resistance mechanisms

**Figure 1:**
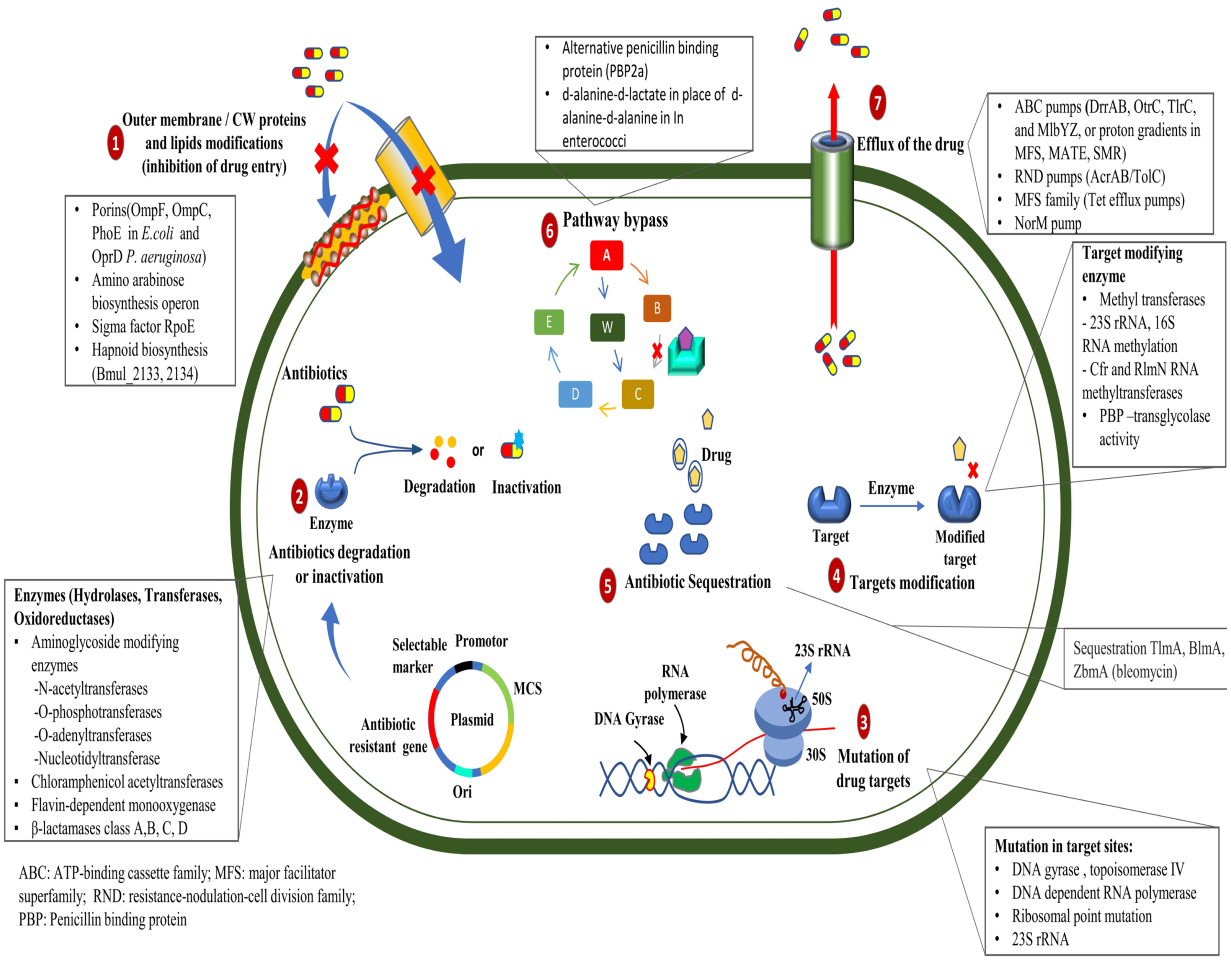
Mechanisms of antibiotic resistance developed by bacteria.

Some of the most important ways (Figure 1) by which bacteria get a survival advantage are as follows:

#### 2.1.1 Mutation in target genes

Mutations in the genes encoding the target proteins of antibiotics can confer resistance in bacteria by changing the structure or function of the target, rendering it less sensitive to the antibiotic’s action (Peterson and Kaur, 2018). The mutations in the *gyrA* and *parC* genes play an essential role in resistance to ciprofloxacin in clinical isolates of *Pseudomonas aeruginosa* (Arabameri et al., 2021). Resistance mechanisms in *Acinetobacter baumannii* include plasmid-associated resistance genes (*qnrA*, *qnrS*, *aac (6′)-Ib-cr*, *oqxA*, and *oqxB*) and chromosomal mutations in the *gyrA* and *parC* genes (Mohammed et al., 2021). In a recent study, the *AmpC* and *AmpR* high expression was associated with resistance to tazobactam, ampicillin, gentamicin, nitrofurantoin, and cephalosporins, whereas *AmpR* deletion reduced β-lactam and aminoglycoside resistance in *Citrobacter freundii* (Tariq et al., 2023).

#### 2.1.2 Efflux pump mutations

The mutation in efflux pumps enables the active elimination of antibiotics from their cellular environment, which can impede the intracellular accumulation of antibiotics in bacterial cells (Whittle et al., 2021). Recent studies have demonstrated that mutations occurring in the *mepA* gene can lead to the development of tigecycline resistance in *Staphylococcus aureus* (Huang et al., 2023). However, mutations and genomic amplifications in the efflux pump gene, *SdrM*, contribute to delafloxacin resistance in methicillin-resistant *S. aureus* (MRSA) (Silva et al., 2023).

#### 2.1.3 Enzyme production

Enzymatic drug resistance manifests by two processes: (a) enzymatic modification of antibiotics and (b) modification of drug targets. The chemical modification of antibiotics by bacterial enzymes renders them ineffective. Aminoglycoside antibiotic resistance is caused by aminoglycoside-modifying enzymes (AME), which alter hydroxyl or amino groups in aminoglycosides, causing them to lose their ability to bind 16S rRNA of the 30S ribosomal subunit (Zárate et al., 2018). In mycobacterial infection, enzymatic inactivation of rifamycin is facilitated by many enzymatic modifications, including ADP ribosyltransferases, glycosyltransferases, phosphotransferases, and monooxygenases (Surette et al., 2021). New Delhi metallo-β-lactamase (NDM-1) is a carbapenemase-producing bacterium having a mutated gene, blaNDM-1, which confers resistance to carbapenems in Enterobacteriaceae and various other bacteria (Khan et al., 2017). Antibiotic resistance, primarily caused by β-lactamase, is prevalent within ESKAPE pathogens (*Enterococcus faecium*, *Staphylococcus aureus*, *Klebsiella pneumoniae*, *Acinetobacter baumannii*, *Pseudomonas aeruginosa*, and *Enterobacter* species), causing significant economic burden and fatality risks (Mancuso et al., 2021). Given their significant implications for global health care, Behzadi et al. (2020) reviewed the distinctive characteristics of metallo-β-lactamases found in microbial pathogens, particularly within the Enterobacteriaceae family. Bacteria can evolve and adapt to antibiotics through modifications to the molecules or structures that are normally targeted. The Erm methyltransferase family alters a nucleotide in the 23S rRNA of the bacterial 50S ribosomal subunit, causing resistance to the prototypic macrolide erythromycin (Weisblum, 1995). The other drug linezolid target, the 23S rRNA region in the 50S ribosomal subunit, was altered by the inactivation of a methyltransferase (Long and Vester, 2011); likewise, the 16S rRNA methylase (ArmA) in *A. baumannii*, methylates adenine residues in the bacterial ribosome, reducing the binding affinity of aminoglycosides such as gentamicin and kanamycin (Jouybari et al., 2021).

#### 2.1.4 Altered antibiotic entry

Mutations in bacterial outer membrane proteins can reduce the permeability of the cell membrane, limiting the entry of antibiotics into the bacterial cell. Increased resistance to penicillin and tetracycline in *Neisseria gonorrhoeae* is due to mutations in the outer membrane protein porin IB at positions G120D and A121D (Olesky et al., 2002). Physical modification in the membrane creates a physical obstruction that hinders the absorption of drugs into the cellular compartment. The modification in the core oligosaccharide of lipopolysaccharide in the outer membrane of *E. coli* inhibits vancomycin action (Stokes et al., 2016).

#### 2.1.5 Horizontal gene transfer

Horizontal gene transfer (HGT) involves organisms transferring genes between themselves, different species or genera, or even across different domains of life in a manner other than traditional reproduction. Many organisms can acquire new genes that can confer advantageous traits in bacteria, such as antibiotic resistance, adaptability, and metabolic versatility, and this mechanism plays a crucial role in their evolution (von Wintersdorff et al., 2016). Bacteria can acquire resistance genes through HGT mechanisms such as conjugation, transformation, or transduction. These resistance genes may be on mobile genetic elements such as plasmids or integrons (Bello-López et al., 2019). HGT occurs more frequently in biofilms than in planktonic cultures, promoting the rapid dissemination of antibiotic-resistance genes (Michaelis and Grohmann, 2023). Outer membrane vesicles can mediate the horizontal transfer of virulence and resistance plasmid *phvK2115* between *Klebsiella pneumoniae* strains and between *K. pneumoniae* and *Escherichia coli* strains (Wang et al., 2022). The vancomycin resistance gene, *vanP*, was presumed to be acquired by HGT from *Clostridium scidens* and *Roseburia* sp. Four hundred and ninety-nine in the *Enterococcus faecium* isolate (Xavier et al., 2021). *A. baumannii* employs HGT to efficiently acquire and exchange mobile genetic elements, contributing to its adaptability. It utilized outer membrane vesicles and phages as transfer mechanisms, thus aiding in the spread of antibiotic resistance genes. Its diverse virulence factors and flexible genome present a significant challenge to global public health systems (Karampatakis et al., 2024).

#### 2.1.6 Antibiotic sequestration

Bacteria can resist antibiotics by sequestering them, a process where drug-binding proteins prevent the antibiotic from reaching its target. These proteins deactivate antibiotics through hydrolysis or chemical modification (Blair et al., 2014). The drug-binding protein AlbA binds to albicidin and confers resistance to *Klebsiella oxytoca* (Rostock et al., 2018). The bleomycin family of antibiotics exhibits resistance in *Streptomyces verticillus* and *Streptoalloteichus hindustanus* strains that produce N-acetyltransferase and a binding protein. The N-acetyltransferase disrupts the antibiotic’s metal-binding domain, while the binding protein sequesters the metal-bound antibiotic and inhibits drug activation (Rudolf et al., 2015).

#### 2.1.7 Biofilm-associated resistance

Biofilms are structured communities of bacteria showing resilience against antibiotics and diverse environmental pressures enclosed within a self-generated matrix composed of polysaccharides, proteins, and DNA (Shree et al., 2023). Biofilms resist antibiotics by utilizing extracellular components such as DNA, enzymes, and regulated genes. This resistance varies depending on the specific antibiotic, making biofilms a major contributor to chronic infections (Bano et al., 2023). Bacteria possess a strong quorum-sensing network system that can respond easily to environmental stress factors (Zhao et al., 2020). The presence of high-density colony populations has been seen to give rise to the production of small molecule signals called autoinducers (Waters and Bassler, 2005; Melke et al., 2010). This network system exhibits successful microbial interaction and physiological processing, constituting one of the best examples of antimicrobial resistance (Prestinaci et al., 2015). The significance of quorum-sensing systems in governing microbial resistance mechanisms, including drug efflux pump regulation and microbial biofilm formation (Zhao et al., 2020). According to statistical data from the National Institute of Health (NIH), biofilm development is observed in roughly 65% of bacterial infections and around 80% of chronic illnesses (Preda and Săndulescu, 2019).

Intraspecies communication regulates cellular functions such as pathogenesis, genetic material transfer, nutrition uptake, and secondary metabolite formation (Kamaruzzaman et al., 2018). This communication is pivotal for the simultaneous development of biofilms in gram-positive bacteria (e.g., *S. aureus*, *S. epidermidis*, and *L. monocytogenes*), which use oligopeptides as signaling molecules (Chen et al., 2016; Zhou et al., 2020). However, gram-negative bacteria (e.g., *P. aeruginosa*, *V. fischeri*, *S. marcescens*, *K. pneumoniae*) utilize N-acyl homoserine lactones (AHLs) as the signaling molecules in this particular system (Steindler and Venturi, 2007; Galloway et al., 2010).

In a cohort study involving *S. aureus*, the samples displayed penicillin resistance, with the majority exhibiting MDR. *In vitro* assessments revealed substantial biofilm production, with approximately one-fourth of the isolates demonstrating these capabilities (Dash et al., 2023). A study revealed that the overexpression of the TaPLA2 constructs in *T. asahii* resulted in increased resistance to azoles, achieved through drug efflux augmentation and biofilm formation (Ma et al., 2023). PatA facilitates mycolic acid production via an unidentified mechanism in *M. tuberculosis*, mitigating the inhibitory effects of isoniazid. Furthermore, PatA was shown to influence biofilm formation and the ability of organisms to withstand environmental stress by modulating lipid production (Wang et al., 2023). Naziri and Majlesi (2022) examined the incidence, patterns of antimicrobial resistance, and biofilm development of methicillin-resistant *S. pseudintermedius* (MRSP) on pets’ skin, exploring the potential for zoonotic transmission. Treating infections caused by these resilient microorganisms can be prolonged and challenging to eradicate. Their presence complicates treatment and management strategies, leading to prolonged illness, increased healthcare costs, and increased patient risk. Additionally, biofilms pose a significant global concern in relation to chronic diseases and medical devices.

##### 2.1.7.1 Association of biofilm with chronic diseases

Chronic diseases are associated with the development of biofilms, which are crucial survival strategies for bacteria. There are a variety of chronic illnesses in which bacteria can form complex biofilms, including chronic wounds, cystic fibrosis, otitis, urinary tract infections (UTIs), and others (Mirzaei et al., 2020). Biofilm formation by gram-negative bacteria worsens chronic and nosocomial infections, particularly chronic respiratory infections. Alternative therapies, such as antimicrobial peptides and liposomal formulations, are becoming increasingly important due to antibiotic resistance (Karmakar et al., 2023). According to experimental research, biofilms are present in chronic wounds at rates ranging from 20 to 100%, indicating their importance in healing (Goswami et al., 2023). Using a dynamic system and a chronic wound-like medium, Pouget et al. (2022) examined the formation and evolution of biofilms formed by *S. aureus* and *P. aeruginosa* in chronic wounds. These bacteria can form robust biofilms, perpetuate chronic infection, impair wound healing, and increase antibiotic resistance (Roy et al., 2020; Qin et al., 2022). The Lubbock chronic wound biofilm model resembling a chronic wound was developed as an *in vitro* study tool to investigate wound healing processes, biofilm inhibition, and the antibacterial efficacy of novel compounds (Diban et al., 2023). Meanwhile, El Masry et al. (2023) also established the swine model, enabling the study of wound biofilm infections by involving the host immune system and monitoring iterative changes during biofilm formation. Antiseptic therapy, with a specific focus on povidone-iodine (Alves et al., 2020) and synthetic antimicrobial peptides, noted for their increased efficacy and reduced toxicity (Pfalzgraff et al., 2018), is employed in the management of chronic wounds and biofilms.

Cystic fibrosis (CF) lung disease is predominantly an infectious condition where robust inflammation prevents the effective elimination of pathogens, hampers the lungs’ function, and results in respiratory failure and death (Cantin et al., 2015). CF patients with chronic *P. aeruginosa* infections produce mucoid alginate and form biofilms, conferring antibiotic resistance and immune responses (Høiby, 2002). Individuals with CF are primarily affected by *P. aeruginosa* and *B. cenocepacia*, where low iron concentrations induce free-living forms and motility. In contrast, high iron concentrations promote aggregation and biofilm formation (Berlutti et al., 2005). As an adjunctive therapy, cephalosporin effectively disperses biofilms formed by *P. aeruginosa* and may benefit patients with CF (Soren et al., 2020).

Urinary tract infections (UTIs) are among humans’ most prevalent bacterial infections, accounting for approximately 40% of all hospital-acquired infections (Haque et al., 2018). Approximately 75% of urinary tract infections acquired in hospital settings are associated with urinary catheters (Al-Qahtani et al., 2019). The pathogenic strains of *E. coli* cause UTIs and can form biofilms that facilitate the bacteria’s survival and persistence. Moreover, these *E. coli* strains possess strong biofilm-forming abilities and are resistant to many antimicrobial agents, including ampicillin, cefazolin, cefepime, ampicillin-sulbactam, and ceftazidime (Karigoudar et al., 2019; Katongole et al., 2020; Ramírez Castillo et al., 2023). A study in western Saudi Arabia involved testing urine samples for *E. coli* prevalence associated with UTIs, with a higher occurrence among females. Among these samples, numerous isolates showed resistance to norfloxacin and ampicillin, with no evidence of biofilm formation detected (Arafa et al., 2022). Another investigation in Ahvaz, Iran, focused on biofilm formation, structural characteristics, and antibiotic resistance of *S. saprophyticus* strains that cause female UTIs. Most *S. saprophyticus* isolates were resistant to erythromycin, with 58% exhibiting MDR. Additionally, 65% of these isolates demonstrated biofilm formation, primarily characterized by a polysaccharide matrix (Hashemzadeh et al., 2020).

Otitis media with effusion (OME), a childhood condition attributed to bacterial infection associated with biofilms, has been found to contain coagulase-negative staphylococci in samples (Daniel et al., 2012). Furthermore, other pathogenic bacteria, such as *H. influenzae*, *S. pneumoniae*, and *M. catarrhalis*, have been reported in infections (Van Hoecke et al., 2016; Korona-Glowniak et al., 2020).

##### 2.1.7.2 Formation of biofilm on medical devices

Utilizing biomaterials such as prosthetic heart valves, intravenous central line prosthetics, contact lenses, urinary tract catheters, and prosthetic joints has been associated with the formation of biofilms, leading to potential infections (Zhao et al., 2013; Li P. et al., 2023). Both gram-positive (*S. aureus*, *E. faecalis*, *S. viridans*, and S. *epidermidis*) and gram-negative bacteria (*P. aeruginosa*, *E. coli*, *P. mirabilis*, and *K. pneumoniae*) can form biofilms on medical devices (Donlan, 2001). Biofilm-associated infections are primarily caused by *S. aureus* and *S. epidermidis*, which are frequently found on cardiovascular devices. Their versatility allows them to transition from single free-floating cells to multicellular biofilms (Schilcher and Horswill, 2020). It is noted that *S. aureus* and *S. epidermidis* are responsible for approximately 40–50% of infections associated with prosthetic heart valves and 50–70% with catheter biofilms (Chen et al., 2013).

Microbial colonization of central venous catheters (CVC) can lead to biofilm formation, aiding bacterial survival against antimicrobial agents and the host immune system, potentially causing severe infections, and spreading to other body sites (Gominet et al., 2017). High-dose antibiotics inside the catheter can significantly reduce bloodstream infection (Wolcott, 2021). A systematic review conducted by Cangui-Panchi et al. (2022) reported that biofilm formation was observed in 59 to 100% of clinical isolates, with prevalence rates varying notably among regions. Various microorganisms were identified among the clinical isolates, including gram-positive and gram-negative strains and *C. albicans*. The findings highlight the association between the high prevalence of biofilm-forming microorganisms and the increased incidence of nosocomial infections among catheterized patients.

The development of mature biofilms on the contact lens surface is associated with severe eye infections such as keratitis. Among different pathogens, *S. aureus* (including MRSA) and *P. aeruginosa* are the most commonly encountered in contact lens-related eye infections (Dosler et al., 2020). Additional fungal pathogens such as *Candida*, *Fusarium*, and *Aspergillus* contribute to the development of keratitis in individuals wearing soft contact lenses, playing a role in contact lens-associated fungal keratitis (Yi et al., 2023). Raksha et al. (2020) collected 265 gram-positive and gram-negative isolates from contact lens wearers and confirmed the presence of biofilm by tube and Congo red agar method.

A catheter-associated urinary tract infection poses significant risks to patients and the healthcare system. In a study, biofilms formed by *E. coli*, *P. aeruginosa*, and *P. mirabilis* were detected on three distinct types of commercially available catheters: hydrogel latex, silicone, and silver alloy-coated hydrogel latex (Wilks et al., 2021). As urease-producing species such as *P. mirabilis* colonize catheter surfaces, they form crystalline biofilms that encrust and block catheter surfaces, resulting in severe clinical complications and necessitating emergency hospital referrals (Pelling et al., 2019). *In vitro* tests showed that urinary catheters containing a blend of rifampicin, sparfloxacin, and triclosan were effective in preventing colonization by common uropathogens, including *S. aureus*, *P. mirabilis*, and *E. coli* (Fisher et al., 2015). Almalki and Varghese (2020) conducted antibiotic sensitivity tests on clinical samples from catheter-associated urinary tract infections (UTIs). *E. coli* was identified as MDR to pan-drug resistant (PDR), while *Klebsiella* and *Pseudomonas* were categorized as extensively drug-resistant (XDR) organisms. However, other isolates such as *E. fecalis*, *S. aureus*, *P. mirabilis*, and *Citrobacter* exhibited resistance to a limited range of antibiotics. Using urine samples and urinary catheter segments, Ramadan et al. (2021) assessed biofilm development using the tube method (TM) and scanning electron microscope (SEM). Their findings revealed an 82.85% prevalence of biofilm-dependent catheter-associated urinary tract infections, with *K. pneumoniae* displaying the highest biofilm-forming capacity.

A significant challenge remains in treating prosthetic joint infections (PJIs), mainly due to the formation of biofilms by infectious bacteria (Gbejuade et al., 2014). During the implantation of a device, an immunologically vulnerable area is created around the device. In this region, the host may be unable to effectively eliminate bacteria, leading to the formation of biofilms on the surface of the biomaterial (Rochford et al., 2012). Sadovskaya et al. (2006) found that biofilm-producing staphylococci isolated from infected orthopedic implants contain two carbohydrate molecules (N-acetyl-D-glucosamine and teichoic acid). Svensson Malchau et al. (2021) characterized the biofilm capabilities and antimicrobial susceptibilities of staphylococci responsible for causing PJIs. They revealed a noteworthy correlation between biofilm formation, increased antimicrobial resistance, and the recurrence of PJIs. Macias-Valcayo et al. (2022) investigated the antimicrobial susceptibility of clinical isolates of gram-negative bacilli from PJIs. Additionally, they examined the possible correlation between antimicrobial resistance and the formation of biofilms.

## 3 Nanoantibiotics

**Figure 2:**
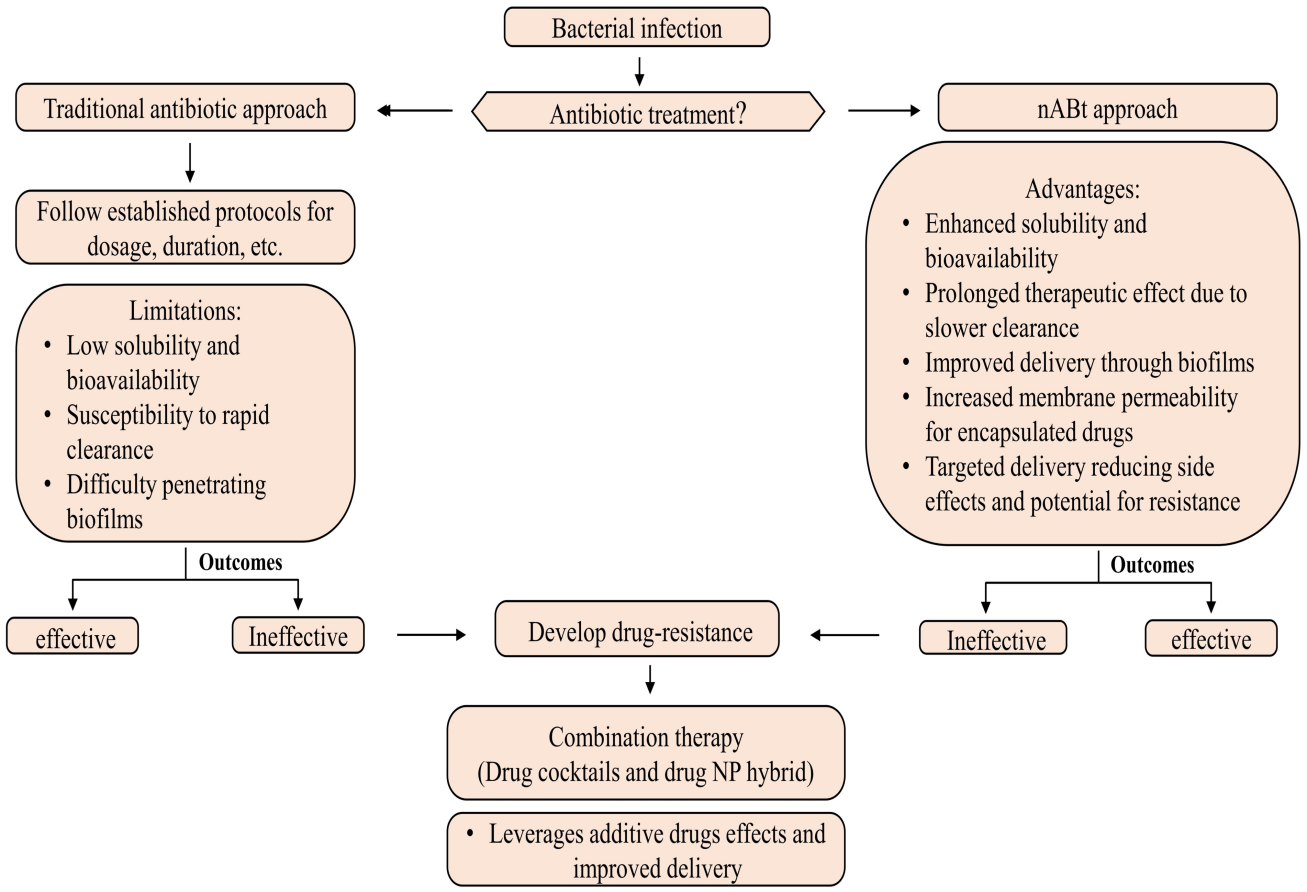
Methods for controlling microbial infections.

nAbts utilize nanoscale vehicles called nanoparticles (NPs) to encapsulate naturally produced and artificially derived compounds. These cutting-edge technologies are at the forefront of medical advancements (Soares et al., 2018). These structured nanomaterials exhibit enhanced antimicrobial activity and play a crucial role in effectively boosting the efficacy of administered antibiotics in combating infectious diseases (Beyth et al., 2015). As compared to traditional antibiotics, nAbts have many advantages. Firstly, encapsulating drugs in NPs improves their solubility and stability, thus increasing their bioavailability and half-life (Yeh et al., 2020). Furthermore, it prevents rapid renal clearance and enzymatic hydrolysis, facilitating a long-term therapeutic effect (Huo et al., 2022). Secondly, nAbts are capable of circumventing bacterial biofilms, thus bypassing the protective barrier that inhibits conventional antibiotic treatment. This results in the effective delivery of drugs to infected tissues (Karnwal et al., 2023). Additionally, they increase membrane permeability, enhancing encapsulated drugs’ therapeutic efficiency (Liu et al., 2022). The targeted delivery reduces the likelihood of side effects, as well as MDR, by minimizing systemic exposure (Wang et al., 2018). The strategies for managing microbial infections are outlined in Figure 2.

Most nAbts are typically smaller than 100 nm in at least one dimension, reflecting their nanoscale nature. Due to their exceptional size and controllability, NPs are suitable for antimicrobial and intracellular bacterial operations (Huh and Kwon, 2011). The size and shape of NPs influence many factors, such as drug delivery efficiency, biodistribution, and interactions with biological systems (Khan et al., 2019). These nanoscale formulations enable enhanced bioavailability and targeted delivery, making them promising candidates for various medical and therapeutic applications (Kirtane et al., 2021). In general, antibiotics target pathogenic bacteria by inhibiting protein synthesis, degrading cell wall components, interfering with energy production and restoration, and disrupting components across a cell membrane (Kohanski et al., 2010; De Maio et al., 2019). Although antibiotics systematically require multiple doses to be effective. In contrast, nAbts may be effective if only a single, target-specific dose is provided (Vallet-Regí et al., 2007; Li et al., 2015). The emergence of antibiotic-resistant pathogens poses a serious health threat, but NPs may provide potential solutions through their properties as antibacterial agents and their ability to deliver customized antibiotics (Ozdal and Gurkok, 2022). Combining therapy, including drug cocktails and drug-NP hybrids, is emerging as a powerful approach for combating bacterial resistance and enhancing antibiotic effectiveness. Through this strategy, both additive drug effects and improved cellular delivery are leveraged, paving the way for more effective and targeted chemotherapy for infection (Adeniji et al., 2022; Brar et al., 2022).

## 4 Nanoparticles

Traditional antibiotic delivery presents challenges such as low solubility, poor permeability, gastrointestinal instability, and limited antibacterial activity, particularly when given orally (Wu et al., 2020). Antibiotic-conjugated NPs have effectively controlled bacterial infections by enhancing antibiotic uptake, local concentration, and other shortcomings of traditional antibiotics (Jelinkova et al., 2019). *Nanotechnology* introduces two groundbreaking tools, nanobactericides and nanocarriers, revolutionizing antibacterial therapy. These nanostructures, abbreviated as NPs, represent cutting-edge advancements in the field. Nanobactericides, tiny warriors with built-in antibacterial properties, attack and destroy microbes directly. In contrast, nanocarriers, discrete transporters, are capable of delivering conventional antibiotics directly to their targets, allowing them to unleash their potent effects within the core of the microbial threat (Vassallo et al., 2020). Given the potential toxicity of many engineered NPs, it is crucial to investigate methods for creating safe NPs, such as those obtained from plant sources.

NPs derived from natural sources exhibit unique properties that make them suitable for use in the antimicrobial field. The rapidity, safety, and cost-effectiveness of synthesizing NPs using plant extracts are characterized by minimal energy consumption and non-toxic derivatives (Patra et al., 2018; Nguyen et al., 2022). Drug delivery NPs typically range from 10 to 1,000 nm, with at least one dimension falling below 100 nm. The diminutive sizes of NPs and their surface chemistry confer pharmaceutically advantageous characteristics, although they may have associated toxic effects (Yusuf et al., 2023). Moreover, their nanometric size facilitates effective interactions with bacteria, another reason for their nomenclature.

**Figure 3:**
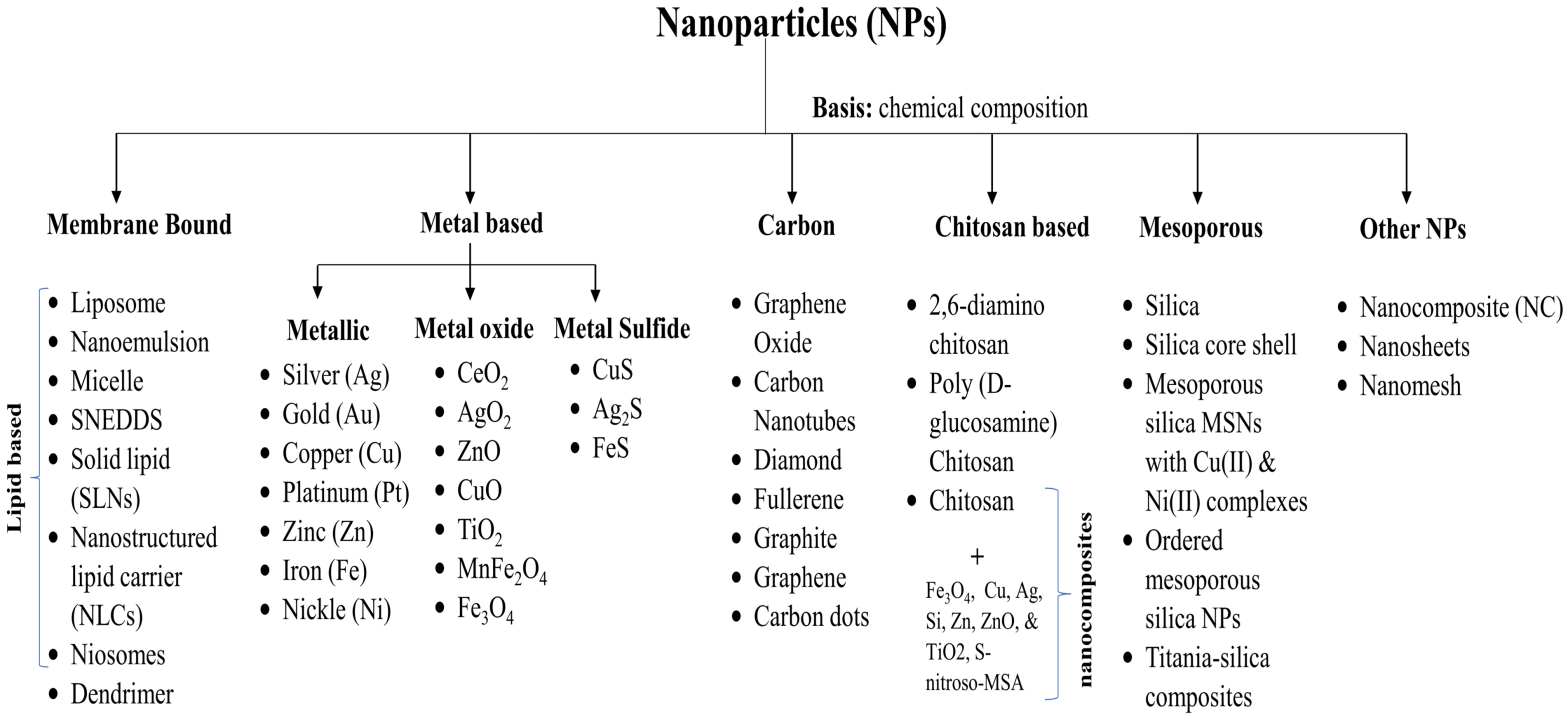
Various categories of nanoparticles utilized for drug delivery.

In general, depending upon the biomolecular conjugation of antibiotics, the NPs have been categorized into different classes, viz. membrane-bound, metal-based, carbon-based, chitosan-based, mesoporous, and others (Figure 3). The membrane-bound or lipid-based NP delivery systems encompass liposomes, self-nano emulsifying drug delivery systems (SNEDDS), solid lipid NPs (SLNs), niosomes, nanostructured lipid carriers (NLCs), and polymeric micelles (Gkartziou et al., 2021). Metal-and metal oxide-based NPs display antibacterial properties due to diverse weak, non-covalent interactions with the ligands and host receptors (Shaikh et al., 2019). NPs derived from metals incorporate heavy metals such as silver (Ag), gold (Au), titanium (Ti), zinc (Zn), iron (Fe), and copper (Cu). Metal oxide NPs, on the other hand, include copper oxide (CuO), cobalt oxide (CoO), titanium oxide (TiO_2_), cerium oxide (CeO_2_), bismuth oxide (Bi_2_O_3_), iron oxide (Fe_2_O_3_), zinc oxide (ZnO), magnesium oxide (MgO_2_), nickel oxide (NiO), etc. (Beyth et al., 2015; Motakef-Kazemi and Yaqoubi, 2020). However, unlike conventional therapies, including radiation or chemotherapy, iron oxide with a hyperthermic effect can be confined to the area containing the magnetic particles, minimizing harm to healthy tissue within the surrounding area (Durmus et al., 2013). Nanocarriers are ubiquitous, but liposomes are the pioneering nanotechnology for this specific application. In addition, dendrimers, cyclodextrins, nanoemulsions, micelles, solid lipid carriers, nanostructured lipid carriers, mesoporous polymeric NPs, hydrogels, fullerenes, and carbon nanotubes are notable nanocarriers (Din et al., 2017; Sultana et al., 2022).

## 5 Structural foundations of nanoantibiotics

In the pharmaceutical industry, nanoformulations have been widely utilized to develop nAbts, leveraging advantages such as enhanced drug loading capacity, prolonged release durations, and better binding affinities (Rana et al., 2019). These nanoscale functionalities have demonstrated the ability to restore drug efficacy in various applications. nAbts exhibit distinctive properties, which allow them to target multiple bacteria concurrently, providing a significant advantage in combating microbial infections (Mamun et al., 2021). A nanoscale antibiotic’s interaction with bacteria has profound implications for the delivery of antibiotics, as nanoscale antibiotics act as drug carriers, penetrate cell membranes, and interfere with protein synthesis in bacteria (Baptista et al., 2018). However, nanomaterials’ effectiveness in targeting bacteria depends upon their physiological state (Gao and Zhang, 2021), including nutrition availability, biofilm formation, bacterial growth stages, and environmental conditions, including aeration, pH, and temperature (Chakraborty et al., 2022). Understanding these complex interactions is essential to designing effective antimicrobial strategies.

An integral aspect of the biology of antibiotics and their associated NPs is their linkage, which can display a diverse array of surface charges such as zwitterionic, cationic, anionic, or neutral (Miller et al., 2015). In nets, the structural and physical characteristics of NPs can be controlled; it is possible to modify the structural characteristics of NPs, such as particle size and lattice constant, increasing charge densities within the NPs and, therefore, increasing the contact area with antibiotics (Mamun et al., 2021). Biomolecularly connected NPs, including metal-ion, oxidative, and non-oxidative components, can interact directly with bacteria (Fasting et al., 2012). Furthermore, nAbts may mitigate the adverse effects of conjugated antibiotics within the host cell (Saha et al., 2007; Gupta et al., 2017). Combining nAbts with pure NP or surface functionalization with structural moieties such as citrate or carboxylate results in prolonged stability to novel antibiotics (Hemaiswarya et al., 2008; Mamun et al., 2021).

## 6 Mechanism of action of nano-bactericides

**Figure 4:**
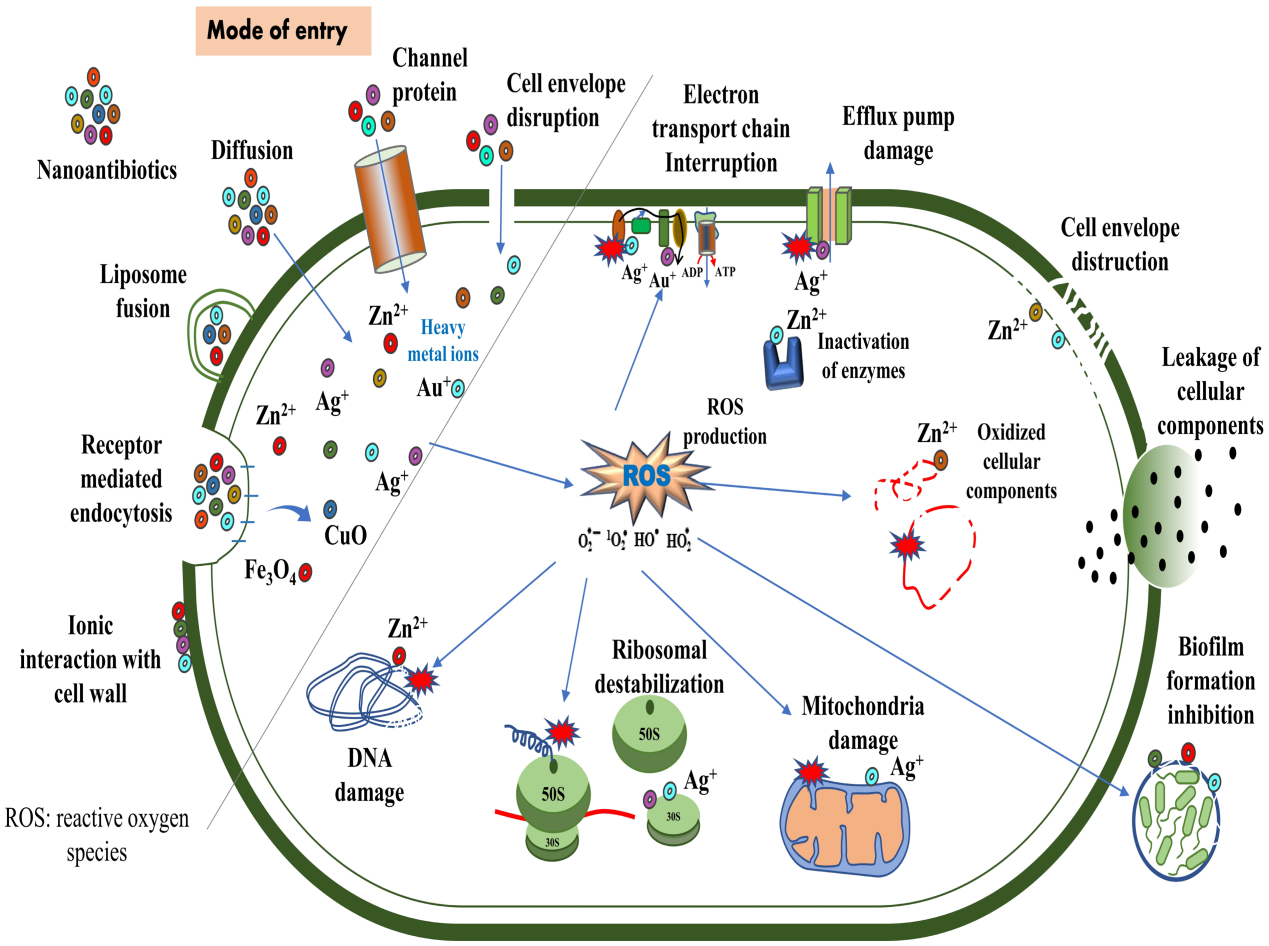
Routes of entry of nanoparticles and their mechanisms of action against bacteria.

The antimicrobial activity of nanomaterials is characterized by physical, chemical, and photo-mediated damage mechanisms. By exploring the interaction between nanomaterials and bacteria, nanotherapeutics may serve as an alternative to traditional antibiotics in treating bacterial infections (Makabenta et al., 2020; Ullah and Khan, 2022). NPs penetrate bacterial envelopes through Van der Waals forces, receptor-ligand interactions, and hydrophobic interactions, damaging the structural integrity of the bacterial membrane. As a result, it interferes with the proton motive force across the cell membrane, limiting the bacteria’s ability to store or produce energy (Erdem et al., 2015). Additionally, it inhibits the enzyme activity of bacteria, suppresses their efflux pumps (Baptista et al., 2018), and increases membrane permeability, which facilitates the accumulation of NPs within membranes and subsequent uptake by cells (Shaikh et al., 2019) (Figure 4). By entering bacterial cells, NPs disrupt microbial pathways, affecting enzymatic proteins, DNA, ribosomes, and lysosomes, resulting in catastrophic outcomes for the bacteria (Karnwal et al., 2023). NPs adhere to the exterior of the biofilm via electrostatic interaction and diffuse throughout the matrix, which is influenced by a variety of factors, including the size, shape, and charge of NPs (Makabenta et al., 2020), the viscosity of exopolysaccharide, cell density, compaction level, liquid flow, and physicochemical interactions with extracellular polymeric substances (Harper et al., 2018; Robino and Scavone, 2020).

According to Liu et al. (2016), titanium alloys infused with copper can effectively eliminate *Streptococcus mutans* and *Porphyromonas gingivalis*. In addition, these alloys prevent the formation of biofilms, thereby reducing bacterial infections and implant failures (Junejo et al., 2023). The binding of gold NP to ATP synthase inhibits ATP synthesis, disrupting energy production. Furthermore, its binding to tRNA inhibits its binding to ribosomes (Cui et al., 2011). Researchers examined the biocidal effects of silver (Ag)-NP by scanning and transmission electron microscope in *E. coli* and observed the pits in the cell wall and accumulation of Ag-NPs (Sondi and Salopek-Sondi, 2004). The copper (Cu)-NP disrupted bacterial membrane integrity, releasing reducing sugars and proteins (Li et al., 2016a). In another study, Cu-NPs strongly inhibited norA efflux pumps by directly binding to the pumps, disrupting efflux kinetics and energy levels (Ashajyothi et al., 2016). Moreover, Au-NP diminished the expression of mexA and mexB genes, reducing active efflux pumps on the cell surface in *P. aeruginosa* (Dorri et al., 2022). Metal oxide NPs, such as titanium dioxide (TiO_2_) and zinc oxide (ZnO), are known to exhibit antimicrobial properties. Upon exposure to light or air, these NPs produce reactive oxygen species (ROS), detrimental to bacterial growth. The ROS can induce oxidative stress, damaging the bacterial cell membrane, DNA, and proteins. This disruption of essential cellular components ultimately leads to the death of the bacteria (Malka et al., 2013; Gold et al., 2018; Prakash et al., 2022). The use of antimicrobial polymers may increase the effectiveness of antimicrobial agents. Nanoengineered antibacterial polymers, with increased surface area and reactivity, have great potential for design and biomedical applications. By inhibiting pathogenic bacteria’s growth or destroying their cell membranes, they possess superior antibacterial activity to conventional agents (Borjihan and Dong, 2020).

## 7 Nanotechnology and nanoparticles in combating drug-resistant strains

Nanotechnology offers a transformative solution to combat the escalating menace of MDR, XDR, and PDR, holding immense promise. To develop innovative strategies for targeted drug delivery, biofilm disruption, and overcoming bacterial resistance mechanisms, researchers are harnessing the unique properties of nanomaterials. The use of nanomaterials has the potential to provide an effective means of combating MDR bacteria due to their diverse antibacterial mechanisms and lower propensity to cause resistance (Li M. et al., 2023). Currently, numerous NPs exhibit *in vitro* antimicrobial efficacy against MDR pathogens, encompassing the ESKAPE pathogen. Moreover, researchers are dedicated to exploring NP pharmacokinetics, pharmacodynamics, and the mechanisms of bacterial resistance (Lee N.-Y. et al., 2019; Adeniji et al., 2022).

Bacterial biofilms contribute to persistent infections by exhibiting increased resistance to antibiotics, disinfectants, and host immune responses (Shree et al., 2023). A promising application of nanotechnology involves the penetration of NPs into biofilms and the exertion of bactericidal effects on those biofilms. Their unique size and characteristics enable these NPs to efficiently target biofilms of drug-resistant pathogens (Hetta et al., 2023). A variety of NPs have been utilized to control microbial biofilm formation. These include metal and metal oxide NPs, solid lipid NPs, liposomes, micro-and nanoemulsions, and polymeric NPs (Mohanta et al., 2023). Metallic NPs represent a promising approach for combating MDR *P. aeruginosa* (Liao et al., 2019; Abeer Mohammed et al., 2022). The conjugation of Ag-NPs with vancomycin demonstrated potent antimicrobial activity against the MDR pathogen (Esmaeillou et al., 2017). Additionally, Ag-NPs synthesized from *Phyllanthus amarus* extract exhibited effective antibacterial potential against MDR strains of *P. aeruginosa* from burn patients (Singh et al., 2014). A study by da Cunha et al. (2023) demonstrated the antibacterial and antibiofilm properties of Ag-NP against MDR *Staphylococcus* species. Additionally, when conjugated with chitosan, Ag-NP demonstrated inhibitory activity against MDR strains of *S. aureus* and *A. baumannii* (Mohammadinejat et al., 2023). The Ag-NPs possess intrinsic antimicrobial properties, whereas the Au-NPs require ampicillin binding to carry out their antimicrobial activity. However, Au-NP and Ag-NP functionalized with ampicillin exhibit broad-spectrum bactericidal activities, especially against MDR bacteria (Brown et al., 2012). Although Ag-NPs have undergone extensive evaluation for their antibacterial properties, there is a scarcity of studies investigating their effectiveness against MDR pathogens, with even fewer addressing XDR or PDR strains.

**Figure 5:**
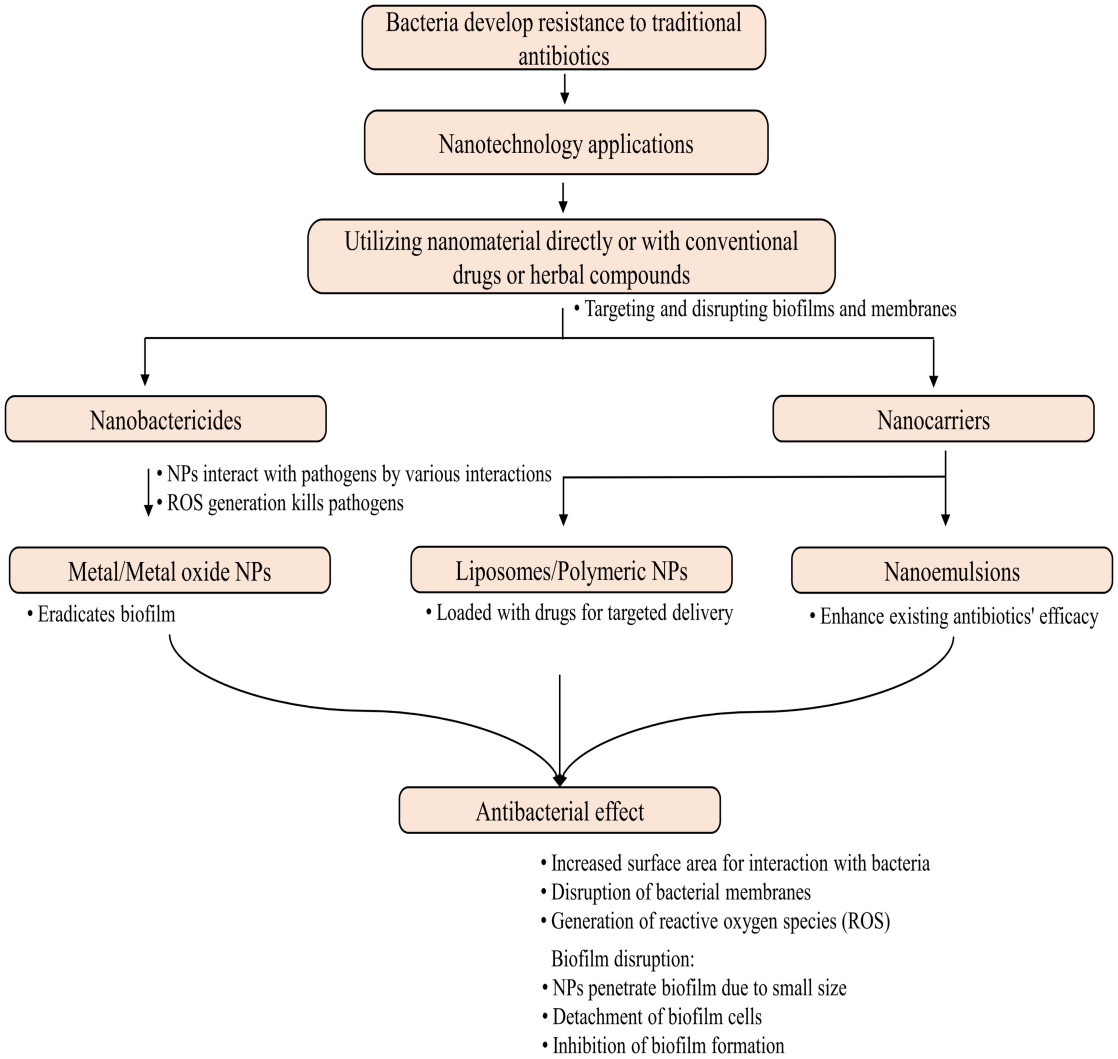
Nanotechnology in combating drug-resistant bacterial infections.

A nanoantibiotic and a SERS-nanoTag have been created by complexing bi-metallic NPs (Au and Ag) with linezolid and 4-mercaptophenyl boronic acid, respectively. These complexes demonstrated effective antibacterial activity against various microorganisms, including MRSA (Hada et al., 2022). Metal oxide NPs, including both ZnO-NPs and a combination of MgO-NPs and ZnO-NPs, exhibit enhanced bactericidal activity against MDR-TB (Yaghubi Kalurazi and Jafari, 2020). A drug-loaded PLGA-NP containing levofloxacin, linezolid, ethambutol, prothionamide, and pyrazinamide has demonstrated promising efficacy and triggers macrophage innate bactericidal events, offering a promising strategy for treating MDR-TB (Jiang et al., 2023). Graphene oxide (GO) serves as an adjuvant for developing improved anti-TB treatments by trapping mycobacteria in the extracellular compartment, thus inhibiting their entry into macrophages (Salustri et al., 2023). Furthermore, the combination of GO with linezolid has been demonstrated to have a potential anti-TB property that is being explored to combat drug-resistant *M. tuberculosis* strains (De Maio et al., 2020). Nanoemulsions containing *Curcuma longa* enhanced ceftazidime’s antibacterial and antibiofilm activity for treating bacterial infections caused by MDR *K. pneumoniae* (Confessor et al., 2024). In another study, NPs loaded with farnesol (FSL NPs) successfully eradicated *S. aureus* within a few hours and achieved 100% inhibition of biofilm formation by drug-resistant *S. aureus* (Maruthapandi et al., 2023). Figure 5 illustrates the contribution of nanotechnology in addressing drug-resistant bacterial infections.

Although nanotechnology has shown promise, its applications against XDRs and PDRs have been addressed in only a few studies where herbal compounds have been explored. The effectiveness of Ag-NPs synthesized from *Helicteres isora* aqueous fruit was evaluated against XDR strains of *P. aeruginosa*. The findings suggest that the disruption of membrane permeability induced by Ag-NPs may account for the growth inhibition and death of the XDR pathogen (Mapara et al., 2015). According to Banihashemi et al. (2021), carbon nanotubes coated with an antibacterial compound have demonstrated antibacterial performance against MDR and XDR strains of *A. baumannii*. However, agar well diffusion and broth microdilution techniques assessed cinnamon oil’s antimicrobial efficacy against XDR and PDR *P. aeruginosa* isolates (Abdelatti et al., 2023). Moreover, another study showed the highest antimicrobial activity against MDR or PDR *H. pylori* strains (Ali et al., 2022). These findings underscore the promising role of nanotechnology in addressing the growing challenge of antimicrobial resistance.

## 8 Plant-derived nanoparticles

Green NP synthesis involves natural materials and environmentally friendly methods, which eliminate the use of harsh chemicals and solvents. NPs created through this method are known for their biocompatibility, sustainability, minimal environmental impact, and cost-effectiveness (Singh et al., 2023). In general, natural NPs are found to have more stability and compatibility than artificial ones due to the presence of capping layers (Javed et al., 2020). Fe-NPs derived from blueberry leaf extracts possess a natural capping of polyphenols that promote stability (Manquián-Cerda et al., 2017). Additionally, these capping layers provide surface area for biological interactions (Singh et al., 2018) and increase the shelf life of the NPs, as well as enhance their physical and biological properties, making them more effective in treating diseases.

Various studies have explored the synthesis of metal and metal oxide NPs, including silver (Ag), gold (Au), copper (Cu), zinc oxide (ZnO), and others, using plant extracts such as *Phyllanthus emblica*, *Trachyspermum ammi*, *Clerodendrum inerme*, *Azadirachta indica*, *Emblica officinalis*, and others. The synthesis of Ag-NP and Au-NP remains challenging due to high energy and chemical requirements, as well as byproduct formation. However, plant-based NPs have medical potential and compatibility for the treatment of drug-resistant microbes (Hammami et al., 2021; Wahab et al., 2021; Balaji et al., 2023). Specifically, Ag-NP derived from *Phyllanthus emblica* fruit extract exhibited significant antimicrobial activity against *Acidovorax oryzae* strain RS-2 (Masum et al., 2019), Au and Ag-NPs from *Clerodendrum inerme* leaf extract (Khan et al., 2020), and Au-NPs synthesized from *Trachyspermum ammi* seed extract effectively targeted drug-resistant biofilms of *Listeria monocytogenes* and *Serratia marcescens* (Perveen et al., 2021). Yadeta Gemachu and Lealem Birhanu (2024) synthesized ZnO, CuO, and NiO-NPs from *Azadirachta indica* leaf extract, with CuO-NPs displaying excellent photocatalytic activity. ZnO-NPs exhibit various unique mechanical attributes, including high catalytic and photochemical activity, a low melting point as biosensors, and exceptional antibacterial and antifungal properties (Sirelkhatim et al., 2015). ZnO-NP from *Emblica officinalis* showed antibacterial and anti-biofilm activity (Kaur et al., 2020), whereas those derived from orange fruit peel extract demonstrated bactericidal activity (Doan Thi et al., 2020). Furthermore, ZnO-NP from the aqueous extract of *Ocimum lamifolium* (Tilahun et al., 2023) and *Cocos nucifera* leaf (Rahman et al., 2022) extract was tested for electrocatalytic activity and photocatalytic activity, respectively, as well as for antimicrobial activities. Similarly, CuO-NPs synthesized from a variety of plant component extracts such as *Catha edulis* leaves (Kelele et al., 2019; Andualem et al., 2020), *Passiflora edulis* leaves (Yasin et al., 2022), *Parthenium hysterophorus* (Nzilu et al., 2023), *Phyllanthus amarus* leaves, *Hibiscus cannabinus* flowers (Kalaiyan et al., 2020), *Piper betle* leaves (Ahmad et al., 2024), and *Piper nigrum* leaves (Naaz et al., 2023). Additionally, other NPs, such as Ni and NiO from *Phytolacca dodecandra* L’Herit leaf (Firisa et al., 2022), MnO_2_ from *Psidium guajava* (Karthik et al., 2024), *Viola betonicifolia* leaf (Lu et al., 2021), and CeO_2_ from *Abelmoschus esculentus* (Ahmed et al., 2021), along with titanium (Ti), palladium (Pd), and platinum (Pt) (Jadoun et al., 2021), have also been explored. As natural antioxidants, these NPs can be used as anticancer, antibacterial, and photocatalytic disinfection agents.

## 9 Herbal compounds as antimicrobials

Herbal compounds possess antimicrobial activity that has unique properties and functions. The defense mechanism of plants lies in the synthesis of a variety of chemical compounds called secondary metabolites (phenols, polyphenols, alkaloids, lectins, terpenoids, essential oils, and others), each of which plays a specific and distinct role (Othman et al., 2019; Al-Khayri et al., 2023). These compounds protect plants against pathogens and other invaders, and they also possess a variety of medicinal properties for humans. They are used to produce various pharmaceutical drugs for treating numerous ailments, such as cancer, diabetes, heart disease, and microbial infections (Wink, 2015; Parham et al., 2020). In medicine, herbal compounds are being transformed into nanoformulations, providing a promising avenue for developing advanced treatments for microbial infections by offering therapeutic efficacy and targeted and controlled delivery methods (Patra et al., 2018). When encapsulated within drug delivery systems, these compounds target drug delivery to specific body regions, enhancing stability and bioavailability and preventing deterioration or evaporation of volatile components. Techniques such as emulsion phase separation, emulsification/internal gelation, and spray drying, among others, are utilized to encapsulate bioactive ingredients effectively (Ozkan et al., 2024).

Ingenious screening techniques will discover medicinal compounds from various herb extracts and oils (Savoia, 2012). The major challenges with phenolic compounds and essential oils (EOs) are their bioaccessibility and bioavailability, which depend on their structure, how they interact with other food components, the quality of the material used to encapsulate them, and how they are encapsulated (Grgić et al., 2020; Lammari et al., 2020). Polyphenolic compounds (flavonoids, tannins, phenols, phenolic acids, flavonoids, quinines, coumarins, and others) possess antioxidant and antimicrobial properties and are used as food additives (Singh et al., 2022), as well as promising new element sources for pharmaceutical and medicinal research (Tungmunnithum et al., 2018). Phenolic extracts from various herbal sources were analyzed for their antioxidant and antibacterial activities against bacteria and fungi (Abdul Qadir et al., 2017). The leaves and flowers of *Ruta chalepensis* L. contain high amounts of polyphenols, flavonoids, and tannins containing vanillic acid and coumarin, which were the most effective against *Pseudomonas aeruginosa* (Ouerghemmi et al., 2016). Phytochemical analysis of phenols and flavonoids from three medicinal herbs, *Achillea millefolium*, *Bergenia ciliata*, and *Aloe barbadensis miller*, indicated that they had antibacterial activity against *Staphylococcus aureus* and *Escherichia coli* (Mehmood et al., 2022). Using the quantitative structure-activity relationship (QSAR) model, Bouarab-Chibane et al. (2019) demonstrated the antibacterial activity of 35 polyphenols against gram-positive and negative bacteria.

Essential oils (EOs) are one of the most extensive classes of herb-based specialized metabolites that play a key role in the plant’s defense response against microbial infections. They have antibacterial, antioxidant, anti-inflammatory, and anticancer properties and provide insight into their mechanism of action and pharmaceutical targets (Sharifi-Rad et al., 2017). Eos, such as tea tree oil (Johansen et al., 2020), cardamom oil (Jamil et al., 2016), oregano essential oil (Lu et al., 2018), patchouli oil (Adhavan et al., 2017), eucalyptus oil (Quatrin et al., 2017), and others obtained from various herbal sources possess activity against pathogenic bacteria (gram-positive and negative), fungi, and parasites. Antibiotics combined with herb-based antibacterial substances have demonstrated synergistic benefits due to drug efflux inhibition and alternate modes of action (Seyyedi-Mansour et al., 2022). Combining nanoencapsulated essential oils with antibiotics leverages the synergies between the oils and their components, and the antibiotic’s resistance to multiple antimicrobial agents has been successfully addressed (Chouhan et al., 2017). The comprehensive screening of bioactive compounds originating from herbs as resistance-modifying agents, particularly those that function synergistically with antibiotics, can aid in the elimination of bacterial resistance. Combinatorial trials with the herb extract and the antibiotic are essential for developing an updated model with observable, long-lasting effects in specific plant regions, as contrasted to their parts (Cheesman et al., 2017; Alam et al., 2022). Some of the most significant gram-negative pathogens are also relatively resistant to antibiotics due to the involvement of antibiotic efflux pumps in their non-specific resistance mechanisms. The efficacy of *Thymus maroccanus* and *T. broussonetii* EO in decreasing chloramphenicol resistance, particularly in MDR gram-negative bacteria, was investigated (Fadli et al., 2011). Merghni et al. (2022) examined turpentine nanoemulsion for antibacterial and antibiofilm capabilities against MRSA. Therefore, it is important to examine the potential of such EOs to combat antibiotic resistance (El-Tarabily et al., 2021). Grapefruit EOs, notably their aldehyde-enriched fraction, have anti-inflammatory characteristics, suggesting that they might be used to generate newer nutraceuticals and functional foods treating inflammatory illnesses (Nikolic et al., 2023). Guava (*Psidium guajava*) leaves EOs have antibacterial and anticancer properties, suggesting they might be a natural treatment for mouth infections and cancer (Alam et al., 2023). Furthermore, a recent study explored the antifungal efficacy of five EOs derived from various *Lavandula hybrida* species against 26 fungal strains isolated from dust in North Africa (Donadu et al., 2024). Some of the herbal compounds involved in nAbts formulations are provided in Table 1.

**Table 1:** Nanoformulations derived from plants secondary metabolites and essential oils for antimicrobial action.

S. No.	Scientific name and common name	Compounds extracted from plant components	Nanoformulation	Particle size (nm)	Encapsulation efficiency (%)	Pathogens	Mechanism of action	References
	Secondary metabolites (phenols and polyphenols)							
1.	*Aloe barbadensis miller* (*Aloe vera*)	Anthraquinone; leaves	AQ-CS-PLA anthraquinone (AQ)-coated polymeric NPs, chitosan (CS), and poly (lactic acid) (PLA)	34 nm	N/A	Gram (−): *Escherichia coli*, *Pseudomonas aeruginosa*, *Klebsiella pneumoniae*, *Proteus vulgaris*	Lipid peroxidation and ROS generation	Dhanapal et al. (2014)
2.	Citrus fruits: *Citrus limon* (Lemon), *Citrus sinensis* (Orange), *Citrus aurantiifolia* (Lime)	D-limonenes; fruit	Limonene nanoemulsion integrated with ε-polylysine	12.21–15.65 nm	N/A	Fungi: *Saccharomyces cerevisiae* Gram (−): *Escherichia coli* Gram (+): *Staphylococcus aureus*, *Bacillus subtilis*	Disrupts the cell membrane, proteins, DNA and RNA	Zahi et al. (2016)
3.	*Curcuma longa* (Turmeric)	Curcumin; dried rhizome	Nanocurcumin	2–40 nm	N/A	Fungi: *Penicillium notatum*, *Aspergillus niger* Gram (−): *Escherichia coli*, *Pseudomonas aeruginosa* Gram (+): *Staphylococcus aureus*, *Bacillus subtilis*	Invade inside the bacteria by damaging the cell wall and eventually killing the cell. Nanocurcumin was more effective against gram-positive bacteria	Singh et al. (2011)
			Silane-hydrogel NPs with curcumin	222 ± 14 nm	N/A	Gram (−): *Pseudomonas aeruginosa* Gram (+): methicillin-resistant *Staphylococcus aureus* (MRSA)	Reduce bacterial burden and enhance wound healing in infected burn wounds	Krausz et al. (2015)
			Nanocurcumin	34.0–359.4 nm	N/A	Gram (−): *Escherichia coli*, *Pseudomonas aeruginosa* Gram (+): *Micrococcus luteus*, *Staphylococcus aureus*	Nanocurcumin inhibited bacterial growth at lower doses than curcumin	Adahoun et al. (2016)
4.	*Eugenia caryophyllata* (Clove)	Eugenol; leaves	Solid lipid NP/eugenol/chitosan + ofloxacin	210 nm	33.5% ± 1.9	Gram (−): *Pseudomonas aeruginosa* Gram (+): *Staphylococcus aureus*	Interact with bacterial cell membrane	Rodenak-Kladniew et al. (2019)
			Chitosan nanoemulsion with eugenol	72.05–83.45 nm	80%	*Aspergillus flavus*	Exhibited superior antifungal and aflatoxin B1 inhibitory activity	Das et al. (2021)
5.	*Eugenia caryophyllata* (Clove), *Allium sativum* (Garlic)	Eugenol, garlic oil (Allicin); leaves	Zein NP/eugenol/garlic oil	150 nm	90%	Gram (−): *Edwardsiella tarda*, *Pseudomonas aeruginosa* Gram (+): *Streptococcus iniae*	Inhibits gram-positive and gram-negative bacterial proliferation	Luis et al. (2020)
6.	*Origanum vulgare* (Oregano), *Thymus vulgaris* (Thyme)	Carvacrol; leaves	Micelle with carvacrol and eugenol	10 nm	N/A	Gram (−): *Escherichia coli* O157:H7 Gram (+): *Listeria monocytogenes*	Significant reduction in pathogens biofilm and destruction of cellular components	Pérez-Conesa et al. (2011)
			Solid lipid NPs (SLNs) with propylene glycol monopalmitate (PGMP) and glyceryl monostearate (GMS)-carvacrol NPs	<200 nm	>98%	Gram (−): *Escherichia coli* Gram (+): *Staphylococcus aureus*	Interacts with phospholipid membrane and affects the permeability	He et al. (2019)
			Poly (caprolactone)-carvacrol NPs	198 nm	83.28% ± 3.62	Gram (−): *Pseudomonas aeruginosa* Gram (+): *Staphylococcus aureus*	In an *ex-vivo* wound model, this approach exhibited significant antimicrobial activity	Mir et al. (2020)
			Ovalbumin-carvacrol gel NPs	71.57–179.1 nm	35.05 -91.86%	Gram (−): *Salmonella* sp. Gram (+): *Bacillus cereus*	Antibacterial properties against food-borne pathogens	Rao et al. (2020)
	Essential oils (EOs)							
1.	*Melaleuca alternifolia* (Tea tree)	Tea tree EO; leaves	Inhalable nanoemulsion with tea tree EO	12.5 nm	N/A	Fungi: *Candida albicans* Gram (−): *Escherichia coli*, *Acinetobacter baumannii*, *Klebsiella pneumoniae* Gram (+): *Staphylococcus aureus*	Inhalable nanoemulsion was promising for treating fungal and bacterial pneumonia. Moreover, at low concentrations, it was more effective against fungal pneumonia *in vivo*	Li et al. (2016b)
2.	*Elettaria Cardamomum* (Cardamom)	Cardamom EO; seeds	Chitosan NPs with cardamom EO	50–100 nm	>90%	Gram (−): *Escherichia coli* Gram (+): methicillin-resistant *Staphylococcus aureus* (MRSA)	Demonstrated strong antibacterial activity against beta-lactamase-producing MRSA and MDR-*E. coli*	Jamil et al. (2016)
3.	*Thymus vulgaris* (Thyme)	Oregano EO (carvacrol, thymol, and p-cymene); leaves	Nanoemulsion of hydroxypropyl methylcellulose (HPMC) with oregano EO	221.3 ± 0.80 nm	N/A	Gram (−): *Salmonella typhimurium*	The composite films possess antibacterial activity against pathogens and free radical scavenging activity	Lee J. Y. et al. (2019)
			Nanovesicles (nanoliposomes (L) and nanoarchaeosomes (A))	114.6 ± 6.4 and 129.2 ± 23.0 nm	N/A	Gram (+): methicillin-resistant *Staphylococcus aureus* (MRSA)	Nanoarchaeosomes show better antibiofilm activity agent than nanoliposomes	Perez et al. (2019)
			Nanoemulsion of thyme EO	20–55.2 nm	10%	Fungi: *Aspergillus brasiliensis*, *A. fumigatus* Gram (−): *Escherichia coli Klebsiella oxytoca* Gram (+): *Bacillus cereus*, *Staphylococcus aureus*	Demonstrated antibacterial, antifungal, and anticancer properties	Doghish et al. (2023)
			Nanoemulsion of thyme EO	122.2 ± 1.079 nm	N/A	Gram (−): *Escherichia coli*, *Pseudomonas aeruginosa* Gram (+): *Staphylococcus aureus*, *Bacillus subtilis*	Demonstrated antibacterial and antitumorigenic activities	Nasra et al. (2023)
4.	*Pogostemon cablin*, Wild varieties *P. heyneanus* and *P. plectranthoides* (Patchouli)	Patchouli EO; leaves	Nanoemulsion with patchouli EO	90.1 ± 0.57 nm, 44.9 ± 0.40 nm, 102.3 ± 2.09 nm	N/A	Fungi: *Candida albicans* Gram (−): *Shigella flexneri* Gram (+): *Staphylococcus aureus*, *Streptococcus mutans*	Wild patchouli shows antibacterial activity against gram-positive, gram-negative, and *C. albicans* bacteria	Adhavan et al. (2017)
5.	*Eucalyptus globulus* (Blue gum)	Eucalyptus EO; leaves	Nanoemulsion with eucalyptus EO impregnated into chitosan	9.4 nm	N/A	Gram (+): *Staphylococcus aureus*	Stronger antibacterial action against *S. aureus* as compared to chitosan film alone	Sugumar et al. (2015)
			Nanoemulsion with eucalyptus EO	76 nm	N/A	Fungi: *Candida albicans* Gram (−): *Pseudomonas aeruginosa*	Potent antimicrobial activity to fight against a wide variety of microbes	Quatrin et al. (2017)
			Chitosan NPs with eucalyptus oil and cellulose acetate	48.26 nm	N/A	Gram (+): *Staphylococcus aureus*	A three-fold increase in antimicrobial activity was observed	Elbhnsawi et al. (2023)
6.	*Cinnamomum zeylanicum*, *Cinnamomum cassia* (Cinnamon)	Cinnamon EO; leaves, barks, and root barks	Nanoemulsion with cinnamon EO	101–620 nm	N/A	Gram (−): *Escherichia coli*, *Salmonella typhimurium*, *Vibrio parahaemolyticus* Gram (+): *Staphylococcus aureus*	Smaller cinnamon oil droplets can enter bacterial cells more readily and damage their cell membrane	Chuesiang et al. (2019)
7.	*Eugenia caryophyllata* (Clove)	Clove EO; flower buds	Solid lipid NPs with clove EO	397 ± 10.1 nm, 786.9 ± 11 nm and 506.4 ± 22 nm	70%	Fungi: *Candida albicans* Gram (−): *Pseudomonas aeruginosa* Gram (+): *Staphylococcus aureus*, *Salmonella typhi*	The composition of SLN and cell wall of microorganisms perform effectively and show better antimicrobial activity	Fazly Bazzaz et al. (2018)
8.	*Cymbopogon flexuosus* (Lemongrass)	Lemongrass EO; leaves	Nanoemulsions with *C. flexuosus* EO	>200 nm	N/A	Fungi: *Candida albicans Cryptococcus grubii* Gram (−): *Pseudomonas aeruginosa* Gram (+): *Staphylococcus aureus*	Exhibited a stronger capacity to prevent the adherence of harmful microbes to surfaces, hence preventing the formation of biofilms	da Silva Gündel et al. (2018a)
9.	*Ocimum basilicum* L. (Basil)	Basil EO; Leaves and flowers	Nanoemulsion with basil EO	<200 nm	N/A	Gram (−): *Klebsiella pneumoniae*, *Enterococcus faecalis*, *Salmonella paratyphi* Gram (+): *Staphylococcus aureus* Larva: *Culex quinquefasciatus*	Antioxidant, antibacterial, and larvicidal properties of *O. basilicum* oil were observed, which may be due to secondary metabolites	Sundararajan et al. (2018)
			Nanoemulsion with basil EO	119 ± 1.13 nm	N/A	Fungi: *Candida albicans*, *Candida tropicalis* Gram (−): *Proteus mirabilis*, *Escherichia coli* Gram (+): *Staphylococcus aureus*	In gram (−) bacteria, it works by crossing the hydrophilic channels, while in gram (+), it works by releasing nanometric droplets directly into the action site	da Silva Gündel et al. (2018b)
		Peppermint, cinnamon and lemongrass EOs	Cellulose acetate (CA) with peppermint (PM), cinnamon (CN), and lemongrass (LG) EOs	140–180 nm	N/A (the load capacity of EOs in CA NCs was decreasing as follows: LG > PM > CN)	Fungi: *Candida albicans* Gram (−): *Pseudomonas aeruginosa*, *Escherichia coli* Gram (+): *Staphylococcus aureus*	The nanomaterials showed a good antimicrobial effect against pathogens, planktonic microbial cultures, and biofilms	Liakos et al. (2018)
10.	*Satureja khuzistanica jamzad* (SKJ)	SKJ EO; stems and leaves	Chitosan (CS) with SKJ EO nanogel	571 nm	30.74%	Gram (−): *Escherichia coli*, *Salmonella paratyphi*, *S. enterica*, *S. typhi*, *Pseudomonas aeruginosa*	CS-SKJ nanogel formulation showed activity against gram-positive and the majority of gram-negative bacteria. Also, the anti-tumor effect was on the KB-cell line	Rashidipour et al. (2021)
11.	*Rosmarinus officinalis* (Rosemary)	Rosemary EO; branches and leaves	Multiple lipid NPs with rosemary EO and cefepime	112–124 nm	31–51%	Gram (−): *Pseudomonas aeruginosa*	Encapsulated rosemary EO had significantly higher antibacterial activity	Ben-Khalifa et al. (2021)
12.	*Zanthoxylum Armatum* (Timur) and *Rosmarinus officinalis* (Rosemary)	Timur EO; seeds	Nanoemulgel (carbopol-940), nanoemulsified in Smix and rosemary oil (Tim-Ros-NEG)	139 ± 6.11 nm	N/A	*Candida albicans*	Nanoemulgels containing timur and rosemary oil revealed effective activity against fungus	Noor et al. (2023)
13.	Pine oleoresin	Turpentine EO; wood	Nanoemulsion with turpentine EO	22.52 nm–26.54 nm	N/A	Gram (+): methicillin-resistant *Staphylococcus aureus* (MRSA)	Significant antibiofilm action against MRSA strains, disrupting around 70.83% of biofilm	Merghni et al. (2022)

## 10 Side effects of herbal compounds

In recent years, herbal medicines have become more prominent in global healthcare. However, their limited solubility, bioavailability, pharmacological activity, and susceptibility to physical and chemical instability and degradation limit their clinical utility (Başaran et al., 2021). These products may contain substances that can either induce or inhibit enzymes involved in drug metabolism. Consequently, the use of drugs and certain medicinal plants may cause severe effects and may diminish therapeutic effectiveness (Singh and Zhao, 2017). Various factors can contribute to the toxicity of herbal medicine products, including plant components or metabolites with a toxic potential, heavy metals, adulteration, pesticides, and fungi or microorganism contamination (Jitareanu et al., 2022).

In contrast to conventional drugs, herbal supplements are not subject to premarketing purity and potency regulations by the US Food and Drug Administration. Herbal supplements can have adverse pharmacological and toxicological effects, including abnormal laboratory results, allergic reactions, genotoxicity, carcinogenicity, teratogenicity, organ damage, and even death in some cases. These incidents significantly strained healthcare resources, leading to overcrowded emergency rooms and hospital wards. Patients with high-risk conditions, such as children and geriatrics, breastfeeding mothers, pregnant women, immunocompromised patients, and those undergoing surgery, should be monitored when using herbal supplements (Illamola et al., 2020; Hassen et al., 2022). Certain herbal compounds may negatively impact the digestive system due to the presence of irritants or toxins. Some commonly used natural drugs for osteoporosis treatment have been associated with mild adverse reactions, including skin rash, gastric issues, constipation, irritability, and abnormal urine. Additionally, extracts of *Boswellia serrata* Roxb, *Hedera helix*, and *Perna canaliculus* for the treatment of osteoarthritis have been linked to significant side effects such as upper abdominal pain and unstable movements (Zhou et al., 2022). For centuries, *Ephedra* has been used as a traditional remedy for bronchoconstriction and contains both pseudoephedrine and ephedrine (Hou et al., 2014). Nonetheless, the combination of ephedrine and theophylline may cause insomnia, nervousness, and gastrointestinal discomfort (Weinberger et al., 1975). Aconitum species are commonly used for pain relief and contain several alkaloids (Chan et al., 2021). It has been documented that herbal preparations containing aconitine can result in severe cardiac toxicity, often manifesting as acute myocardial infarction accompanied by chest tightness, ultimately leading to death (Lin et al., 2011). A primary active component of mahuang, ephedrine is known to promote weight loss and modify lipid levels. Nonetheless, it carries the risk of cardiovascular side effects, including a potential rise in heart rate (Yoo et al., 2021).

Long-term use of certain herbal compounds, mainly herbal supplements marketed as “natural” alternatives to prescription medications, can lead to liver and kidney damage (Britza et al., 2022; Lin and Tujios, 2023). Certain significant hepatotoxic herbal supplements, including kava, chaparral, comfrey, coltsfoot, germander, and pennyroyal oil, have been linked to significant liver damage (Dasgupta, 2020). At the same time, several nephrotoxic components are found in herbs, including aristolochic acids and certain alkaloids. Moreover, anthraquinones, flavonoids, and glycosides derived from herbs are recognized as potential kidney toxins (Yang et al., 2018). These side effects are usually associated with excessive use, improper dosage, or herbal compound interactions with prescription medications. Many herbal compounds, especially those used in aromatherapy, have been found to cause dermatitis, phototoxicity, oral toxicity (Farrar and Farrar, 2020), and respiratory issues, including congestion, coughing, and wheezing. Many essential oils were associated with adverse effects, including lavender, tea tree, and peppermint oil (Posadzki et al., 2012). There is no doubt that herbal compounds can be beneficial when used appropriately. However, it is also essential to understand the potential side effects associated with their use.

Combining NPs with herbal medicine in herbal formulation research presents a promising strategy for treating diverse diseases. This approach provides several advantages for herbal drugs, including improved solubility and bioavailability, increased stability, reduced toxicity risks, enhanced pharmacological activity, optimized macrophage distribution, sustained delivery, and protection against physical and chemical degradation. Therefore, nano-sized drug delivery systems for herbal medicines possess the potential to enhance efficacy and address challenges associated with herbal medicines.

## 11 Nanoantibiotics versus microbial infection

The nanoengineered systematic formulations of nAbts, including nanoemulsions, carbon quantum nanodots, fullerene particles, and multi-walled carbon nanotubes, are being studied thoroughly for their target-specific drug delivery patterns, antimicrobial potency, and minimal toxicity values (Debnath and Srivastava, 2021). These nAbts are found to have higher efficacy in terms of drug activation, biocompatibility, the synergistic interaction of NPs for efficient drug delivery, easier drug release strategy at the target site, and structured pathogen killing (Seyyedi-Mansour et al., 2022). The zwitterionic nAbts based on carbon quantum dots facilitate the pathogen removal from the host cells by initiating programmed cell death within the infected bacterial cells (Bing et al., 2016). Similarly, the hydrophobic surfaces of multi-walled carbon nanotube-based nAbts entrap pathogenic phospholipid bilayers, compromising their integrity and promoting cell death (Azizi-Lalabadi et al., 2020).

The use of these nAbts in clinical aspects is categorized into two sections: the associated NPs enhance the functionality of the conjugated antibiotics and, secondly, enable novel bactericidal activities (Chidre et al., 2023). The mechanism for each type of nAbts varies depending upon the conjugated molecule structure, physio-chemical attributes, and their mode of action within targeted pathogenic bacteria cells (Yeh et al., 2020). For instance, vancomycin, one of the glycopeptide antibiotics, has been studied *in vivo* and *in vitro* for antibacterial functions within bacterial cells alongside the synergic formulation of nanostructured systems (Pichavant et al., 2016). As in its non-conjugated form, it was inactive against *Staphylococcus aureus* (Berini et al., 2021). In tackling ongoing infections and chronic wounds, nanocomposites offer a potential alternative to antibiotics, effectively breaking down bacterial biofilms (Varma et al., 2023). Additionally, the combined use of rifampin-infused mussel-inspired silver NPs significantly improves antibacterial performance against MDR strains of *Mycobacterium* (Yu et al., 2021). *In vivo* studies revealed that the nAbts, formulated with rifampicin and SNEDDS, targeted *Mycobacterium bovis* BCG through internalization and intracellular trafficking, along with regulated drug release (Hussain et al., 2019).

In combination with antibiotics, nanomaterials hold immense promise for combating MRD pathogens. They can significantly enhance the effectiveness of traditional antimicrobial agents by overcoming resistance mechanisms. However, several limitations impede the widespread adoption of nanomaterials across various applications. The biggest concern is their potential toxicity, attributed to their diminutive size and unique properties that can result in unpredictable interactions with biological systems. Furthermore, complex and expensive synthesis processes hinder scalability and wide application, while stability issues, including degradation over time, pose challenges for therapeutic applications such as drug delivery (Tang et al., 2022). However, inorganic NPs have some significant limitations, including high dosages, significant toxicity, and limited efficacy, in addition to uncertainty in their long-term safety and biological metabolism (Tsikourkitoudi et al., 2022; Wang et al., 2022). Carbon-based nanomaterials are also toxic and can cause oxidative stress and inflammation (Manke et al., 2013; Wang et al., 2022). Furthermore, the stability of carbon-based organic nanostructures and nanocomposites under various conditions poses a potential concern, particularly for the use of antibiotics (Modi et al., 2022). NP interactions with the immune system have raised concerns, with reports suggesting potential negative outcomes, such as immune stimulation leading to immunosuppression and inflammation, thereby increasing the risk of infection (Yuan et al., 2019). The interaction of nanomaterials with their environment highlights the necessity of thorough evaluation and management of these materials prior to widespread adoption.

## 12 Conclusion and future perspectives

The scientific community has shifted its focus to alternative solutions in response to the increasing number of antibiotic-resistant bacterial strains. Nanoformulations, utilizing the Trojan Horse effect, have emerged as promising candidates. Additionally, nanotechnology is paving the way for the development of effective treatments to combat antimicrobial resistance. Developing nAbts requires extensive interdisciplinary collaboration, combining microbiology with nanomaterial science to achieve specificity, elucidate antibacterial mechanisms, and ensure biosafety. For seamless integration of nAbts into clinical settings, continued refinement of their composition, structure, and pharmacokinetic attributes is essential. Using herbal compounds in combination with traditional antibiotics and nanomaterials or transforming them into nanostructures presents a promising approach to overcoming resistance to bacteria. The inherent antimicrobial properties of essential oils and natural products provide a basis for creating nanoformulations that can be used to combat infections, reduce toxicity, and improve biocompatibility. In addition, nanomaterials disrupt bacterial membranes, inhibit biofilm formation, and enhance the uptake of antibiotics by the cell, ultimately enhancing the effectiveness of antibiotics against resistant bacteria. This synergistic approach harnessing the powers of nanomaterials and antibiotics holds promise for combating MDR and addressing the urgent global health challenge of antibiotic-resistant infections.

The government, healthcare organizations, researchers, and the public must act together to counter alarming projections concerning the impact of AMR in the future. The pursuit of sustainable healthcare solutions requires balancing scientific progress with responsible implementation. The balanced approach, combining innovation with ethical practices, has the potential to effectively address the issue of drug resistance as well as save the lives of many people. A conscientious approach to global health issues is embodied through the synthesis of nature’s bounty and cutting-edge technology and promising improved healthcare outcomes. In summary, *nanotechnology* is a multifaceted field that offers boundless opportunities for exploration and discovery in the world of antimicrobial resistance. The future of combating antimicrobial resistance lies in the convergence of nature and nanotechnology. In this transformative journey, relentless innovation and collaborative efforts will pave the way for a healthier, more resilient global community.

## References

[ref1] Abdelatti M. Abd El-Aziz N. El-Naenaeey Y. Ammar A. Alharbi N. Alharthi A.. (2023). Antibacterial and anti-efflux activities of cinnamon essential oil against pan and extensive drug-resistant *Pseudomonas aeruginosa* isolated from human and animal sources. Antibiotics 12:1514. doi: 10.3390/antibiotics12101514, PMID: 37887215 PMC10604284

[ref2] Abdul Qadir M. Shahzadi S. K. Bashir A. Munir A. Shahzad S. (2017). Evaluation of phenolic compounds and antioxidant and antimicrobial activities of some common herbs. Int. J. Anal. Chem. 2017, 3475738–3475736. doi: 10.1155/2017/3475738, PMID: 28316626 PMC5337800

[ref3] Abeer Mohammed A. B. Abd Elhamid M. M. Khalil M. K. M. Ali A. S. Abbas R. N. (2022). The potential activity of biosynthesized silver nanoparticles of *Pseudomonas aeruginosa* as an antibacterial agent against multidrug-resistant isolates from intensive care unit and anticancer agent. Environ. Sci. Eur. 34:109. doi: 10.1186/s12302-022-00684-2

[ref4] Adahoun M. AL-Akhras M.-A. Jaafar M. Bououdina M. (2016). Enhanced anti-cancer and antimicrobial activities of curcumin nanoparticles. Artif. Cells Nanomed. Biotechnol. 45, 98–107. doi: 10.3109/21691401.2015.1129628, PMID: 26747522

[ref5] Adeniji O. O. Nontongana N. Okoh J. C. Okoh A. I. (2022). The potential of antibiotics and nanomaterial combinations as therapeutic strategies in the management of multidrug-resistant infections: a review. Int. J. Mol. Sci. 23:15038. doi: 10.3390/ijms232315038, PMID: 36499363 PMC9736695

[ref6] Adhavan P. Kaur G. Princy A. Murugan R. (2017). Essential oil nanoemulsions of wild patchouli attenuate multi-drug resistant gram-positive, gram-negative and *Candida albicans*. Ind. Crop. Prod. 100, 106–116. doi: 10.1016/j.indcrop.2017.02.015

[ref7] Ahmad A. Khan M. Osman S. M. Haassan A. M. Javed M. H. Ahmad A.. (2024). Benign-by-design plant extract-mediated preparation of copper oxide nanoparticles for environmentally related applications. Environ. Res. 247:118048. doi: 10.1016/j.envres.2023.118048, PMID: 38160981

[ref8] Ahmed H. E. Iqbal Y. Aziz M. H. Atif M. Batool Z. Hanif A.. (2021). Green synthesis of CeO_2_ nanoparticles from the *Abelmoschus esculentus* extract: evaluation of antioxidant, anticancer, antibacterial, and wound-healing activities. Molecules 26:4659. doi: 10.3390/molecules26154659, PMID: 34361812 PMC8347483

[ref9] Alam M. Bano N. Ahmad T. Sharangi A. B. Upadhyay T. K. Alraey Y.. (2022). Synergistic role of plant extracts and essential oils against multidrug resistance and gram-negative bacterial strains producing extended-spectrum. Antibiotics 11:855. doi: 10.3390/antibiotics11070855, PMID: 35884109 PMC9312036

[ref10] Alam A. Jawaid T. Alsanad S. M. Kamal M. Balaha M. F. (2023). Composition, antibacterial efficacy, and anticancer activity of essential oil extracted from *Psidium guajava* (L.) leaves. Plants 12:246. doi: 10.3390/plants12020246, PMID: 36678958 PMC9863818

[ref11] Algammal A. Hetta H. F. Mabrok M. Behzadi P. (2023). Editorial: Emerging multidrug-resistant bacterial pathogens “superbugs”: a rising public health threat. Front. Microbiol. 14:1135614. doi: 10.3389/fmicb.2023.1135614, PMID: 36819057 PMC9930894

[ref12] Ali S. S. Abd Elnabi M. K. Alkherkhisy M. M. Hasan A. Li F. Khalil M.. (2022). Exploring the potential of *Cinnamomum zeylanicum* oil against drug resistant *Helicobacter pylori*-producing cytotoxic genes. J. Appl. Biomed. 20, 22–36. doi: 10.32725/jab.2022.003, PMID: 35225438

[ref13] Al-Khayri J. M. Rashmi R. Toppo V. Chole P. B. Banadka A. Sudheer W. N.. (2023). Plant secondary metabolites: the weapons for biotic stress management. Metabolites 13:716. doi: 10.3390/metabo13060716, PMID: 37367873 PMC10302943

[ref14] Almalki M. A. Varghese R. (2020). Prevalence of catheter associated biofilm producing bacteria and their antibiotic sensitivity pattern. J. King Saud Univ. Sci. 32, 1427–1433. doi: 10.1016/j.jksus.2019.11.037

[ref15] Al-Qahtani M. Safan A. Jassim G. Abadla S. (2019). Efficacy of anti-microbial catheters in preventing catheter associated urinary tract infections in hospitalized patients: a review on recent updates. J. Infect. Public Health 12, 760–766. doi: 10.1016/j.jiph.2019.09.009, PMID: 31628048

[ref16] Alves P. J. Barreto R. T. Barrois B. M. Gryson L. G. Meaume S. Monstrey S. J. (2020). Update on the role of antiseptics in the management of chronic wounds with critical colonisation and/or biofilm. Int. Wound J. 18, 342–358. doi: 10.1111/iwj.13537, PMID: 33314723 PMC8244012

[ref17] Anand U. Carpena M. Kowalska-Góralska M. Garcia-Perez P. Sunita K. Bontempi E.. (2022). Safer plant-based nanoparticles for combating antibiotic resistance in bacteria: a comprehensive review on its potential applications, recent advances, and future perspective. Sci. Total Environ. 821:153472. doi: 10.1016/j.scitotenv.2022.153472, PMID: 35093375

[ref18] Andrews J. M. (2001). Determination of minimum inhibitory concentrations. J. Antimicrob. Chemother. 48, 5–16. doi: 10.1093/jac/48.suppl_1.5, PMID: 11420333

[ref19] Andualem W. W. Sabir F. K. Mohammed E. T. Belay H. H. Gonfa B. A. (2020). Synthesis of copper oxide nanoparticles using plant leaf extract of *Catha edulis* and its antibacterial activity. J Nanotechnol 2020, 1–10. doi: 10.1155/2020/2932434, PMID: 39136038

[ref20] Antimicrobial Resistance Collaborators (2022). Global burden of bacterial antimicrobial resistance in 2019: a systematic analysis. Lancet 399, 629–655. doi: 10.1016/S0140-6736(21)02724-0, PMID: 35065702 PMC8841637

[ref21] Arabameri N. Heshmatipour Z. Eftekhar Ardebili S. Jafari Bidhendi Z. (2021). The role of gene mutations (gyrA, parC) in resistance to ciprofloxacin in clinical isolates of *Pseudomonas aeruginosa*. Iran. J. Pathol. 16, 426–432. doi: 10.30699/IJP.2021.520570.2542, PMID: 34567192 PMC8463757

[ref22] Arafa S. H. Alshehri W. A. Organji S. R. Elbanna K. Obaid N. A. Aldosari M. S.. (2022). Antimicrobial resistance, virulence factor-encoding genes, and biofilm-forming ability of community-associated uropathogenic *Escherichia coli* in Western Saudi Arabia. Pol. J. Microbiol. 71, 325–339. doi: 10.33073/pjm-2022-029, PMID: 36048880 PMC9608162

[ref23] Ashajyothi C. Harish K. H. Dubey N. Chandrakanth R. K. (2016). Antibiofilm activity of biogenic copper and zinc oxide nanoparticles-antimicrobials collegiate against multiple drug resistant bacteria: a nanoscale approach. J. Nanostructure Chem. 6, 329–341. doi: 10.1007/s40097-016-0205-2

[ref24] Atanasov A. Zotchev S. Dirsch V. Orhan I. Banach M. Rollinger J.. (2021). Natural products in drug discovery: advances and opportunities. Nat. Rev. Drug Discov. 20, 200–216. doi: 10.1038/s41573-020-00114-z, PMID: 33510482 PMC7841765

[ref25] Azizi-Lalabadi M. Hashemi H. Feng J. Jafari S. M. (2020). Carbon nanomaterials against pathogens; the antimicrobial activity of carbon nanotubes, graphene/graphene oxide, fullerenes, and their nanocomposites. Adv. Colloid Interf. Sci. 284:102250. doi: 10.1016/j.cis.2020.102250, PMID: 32966964

[ref26] Balaji V. Perumal S. Palanisamy S. Karuppaiah M. Asaithambi S. Velauthapillai D.. (2023). Bio-inspired synthesis of silver nanoparticles and their nanocomposites for antibacterial and anticancer activity: a comparative study. J. Alloys Compd. 966:171503. doi: 10.1016/j.jallcom.2023.171503

[ref27] Banihashemi K. Amirmozafari N. Mehregan I. Bakhtiari R. Sobouti B. (2021). Antibacterial effect of carbon nanotube containing chemical compounds on drug-resistant isolates of *Acinetobacter baumannii*. Iran. J. Microbiol. 13, 112–120. doi: 10.18502/ijm.v13i1.5501, PMID: 33889370 PMC8043832

[ref28] Bano S. Hassan N. Rafiq M. Hassan F. Rehman M. Iqbal N.. (2023). Biofilms as battlefield armor for bacteria against antibiotics: challenges and combating strategies. Microorganisms 11:2595. doi: 10.3390/microorganisms11102595, PMID: 37894253 PMC10609369

[ref29] Baptista P. V. McCusker M. P. Carvalho A. Ferreira D. A. Mohan N. M. Martins M.. (2018). Nano-strategies to fight multidrug resistant bacteria-“A Battle of the Titans”. Front. Microbiol. 9:1441. doi: 10.3389/fmicb.2018.01441, PMID: 30013539 PMC6036605

[ref30] Barua S. Mitragotri S. (2014). Challenges associated with penetration of nanoparticles across cell and tissue barriers: a review of current status and future prospects. Nano Today 9, 223–243. doi: 10.1016/j.nantod.2014.04.008, PMID: 25132862 PMC4129396

[ref31] Başaran N. Paslı D. Başaran A. A. (2021). Unpredictable adverse effects of herbal products. Food Chem. Toxicol. 159:112762. doi: 10.1016/j.fct.2021.112762, PMID: 34896186

[ref32] Behzadi P. García-Perdomo H. A. Karpiński T. M. Issakhanian L. (2020). Metallo-ß-lactamases: a review. Mol. Biol. Rep. 47, 6281–6294. doi: 10.1007/s11033-020-05651-9, PMID: 32654052

[ref33] Bello-López J. M. Cabrero-Martínez O. A. Ibáñez-Cervantes G. Hernández-Cortez C. Pelcastre-Rodríguez L. I. Gonzalez-Avila L. U.. (2019). Horizontal gene transfer and its association with antibiotic resistance in the genus *Aeromonas* spp. Microorganisms 7:363. doi: 10.3390/microorganisms7090363, PMID: 31540466 PMC6780555

[ref34] Ben-Khalifa R. Gaspar F. B. Pereira C. Chekir-Ghedira L. Rodríguez-Rojo S. (2021). Essential oil and hydrophilic antibiotic co-encapsulation in multiple lipid nanoparticles: proof of concept and *in vitro* activity against *Pseudomonas aeruginosa*. Antibiotics 10:1300. doi: 10.3390/antibiotics10111300, PMID: 34827238 PMC8614727

[ref35] Berini F. Orlandi V. T. Gamberoni F. Martegani E. Armenia I. Gornati R.. (2021). Antimicrobial activity of nanoconjugated glycopeptide antibiotics and their effect on *Staphylococcus aureus* biofilm. Front. Microbiol. 12:657431. doi: 10.3389/fmicb.2021.657431, PMID: 34925248 PMC8674785

[ref36] Berlutti F. Morea C. Battistoni A. Sarli S. Cipriani P. Superti F.. (2005). Iron availability influences aggregation, biofilm, adhesion and invasion of Pseudomonas aeruginosa and *Burkholderia cenocepacia*. Int. J. Immunopathol. Pharmacol. 18, 661–670. doi: 10.1177/039463200501800407, PMID: 16388713

[ref37] Beyth N. Houri-Haddad Y. Domb A. Khan W. Hazan R. (2015). Alternative antimicrobial approach: nano-antimicrobial materials. Evid. Based Complement. Alternat. Med. 2015:246012. doi: 10.1155/2015/246012, PMID: 25861355 PMC4378595

[ref38] Bhatwalkar S. B. Mondal R. Krishna S. B. N. Adam J. K. Govender P. Anupam R. (2021). Antibacterial properties of organosulfur compounds of garlic (*Allium sativum*). Front. Microbiol. 12:613077. doi: 10.3389/fmicb.2021.613077, PMID: 34394014 PMC8362743

[ref39] Bing W. Sun H. Yan Z. Ren J. Qu X. (2016). Programmed bacteria death induced by carbon dots with different surface charge. Small 12, 4713–4718. doi: 10.1002/smll.201600294, PMID: 27027246

[ref40] Binnebose A. Mullis A. Haughney S. Narasimhan B. Bellaire B. (2023). Nanotherapeutic delivery of antibiotic cocktail enhances intra-macrophage killing of *Mycobacterium marinum*. Front. Antibiot. 2:1162941. doi: 10.3389/frabi.2023.1162941 39816663 PMC11732124

[ref41] Blair J. M. A. Webber M. A. Baylay A. J. Ogbolu D. O. Piddock L. J. V. (2014). Molecular mechanisms of antibiotic resistance. Nat. Rev. Microbiol. 13, 42–51. doi: 10.1038/nrmicro3380, PMID: 25435309

[ref42] Bonifácio B. V. da Silva P. B. Ramos M. A. D. S. Negri K. M. S. Bauab T. M. Chorilli M. (2013). Nanotechnology-based drug delivery systems and herbal medicines: a review. Int. J. Nanomedicine 9, 1–15. doi: 10.2147/IJN.S52634, PMID: 24363556 PMC3862741

[ref43] Borjihan Q. Dong A. (2020). Design of nanoengineered antibacterial polymers for biomedical applications. Biomater. Sci. 8, 6867–6882. doi: 10.1039/D0BM00788A, PMID: 32756731

[ref44] Bouarab-Chibane L. Forquet V. Lantéri P. Clément Y. Léonard-Akkari L. Oulahal N.. (2019). Antibacterial properties of polyphenols: characterization and QSAR (quantitative structure-activity relationship) models. Front. Microbiol. 10:829. doi: 10.3389/fmicb.2019.00829, PMID: 31057527 PMC6482321

[ref45] Boucher H. W. Talbot G. H. Bradley J. S. Edwards J. E. Gilbert D. Rice L. B.. (2009). Bad bugs, no drugs: no ESKAPE! An update from the Infectious Diseases Society of America. Clin. Infect. Dis. 48, 1–12. doi: 10.1086/595011, PMID: 19035777

[ref46] Boverhof D. R. Bramante C. M. Butala J. H. Clancy S. F. Lafranconi M. West J.. (2015). Comparative assessment of nanomaterial definitions and safety evaluation considerations. Regul. Toxicol. Pharmacol. 73, 137–150. doi: 10.1016/j.yrtph.2015.06.001, PMID: 26111608

[ref47] Brar A. Majumder S. Navarro M. Z. Benoit-Biancamano M.-O. Ronholm J. George S. (2022). Nanoparticle-enabled combination therapy showed superior activity against multi-drug resistant bacterial pathogens in comparison to free drugs. Nanomaterials 12:2179. doi: 10.3390/nano12132179, PMID: 35808015 PMC9268018

[ref48] Britza S. M. Byard R. W. Musgrave I. F. (2022). Traditional Chinese medicine-associated nephrotoxicity and the importance of herbal interactions—an overview. Pharmacol. Res. Mod. Chin. Med. 3:100099. doi: 10.1016/j.prmcm.2022.100099

[ref49] Brown A. N. Smith K. Samuels T. A. Lu J. Obare S. O. Scott M. E. (2012). Nanoparticles functionalized with ampicillin destroy multiple-antibiotic-resistant isolates of *Pseudomonas aeruginosa* and *Enterobacter aerogenes* and methicillin-resistant *Staphylococcus aureus*. Appl. Environ. Microbiol. 78, 2768–2774. doi: 10.1128/AEM.06513-11, PMID: 22286985 PMC3318834

[ref50] Bush K. Bradford P. A. (2016). β-lactams and β-lactamase inhibitors: an overview. Cold Spring Harb. Perspect. Med. 6:a025247. doi: 10.1101/cshperspect.a025247, PMID: 27329032 PMC4968164

[ref51] Cangui-Panchi S. P. Ñacato-Toapanta A. L. Enríquez-Martínez L. J. Reyes J. Garzon-Chavez D. Machado A. (2022). Biofilm-forming microorganisms causing hospital-acquired infections from intravenous catheter: a systematic review. Curr. Res. Microb. Sci. 3:100175. doi: 10.1016/j.crmicr.2022.100175, PMID: 36518176 PMC9743049

[ref52] Cantin A. M. Hartl D. Konstan M. W. Chmiel J. F. (2015). Inflammation in cystic fibrosis lung disease: pathogenesis and therapy. J. Cyst. Fibros. 14, 419–430. doi: 10.1016/j.jcf.2015.03.003, PMID: 25814049

[ref53] Chakraborty N. Jha D. Roy I. Kumar P. Gaurav S. S. Marimuthu K.. (2022). Nanobiotics against antimicrobial resistance: harnessing the power of nanoscale materials and technologies. J. Nanobiotechnology 20:375. doi: 10.1186/s12951-022-01573-9, PMID: 35953826 PMC9371964

[ref54] Chan Y.-T. Wang N. Feng Y. (2021). The toxicology and detoxification of aconitum: traditional and modern views. Chin. Med. 16:61. doi: 10.1186/s13020-021-00472-9, PMID: 34315520 PMC8314510

[ref55] Cheesman M. J. Ilanko A. Blonk B. Cock I. E. (2017). Developing new antimicrobial therapies: are synergistic combinations of plant extracts/compounds with conventional antibiotics the solution? Pharmacogn. Rev. 11, 57–72. doi: 10.4103/phrev.phrev_21_17, PMID: 28989242 PMC5628525

[ref56] Chen Y. Liu T. Wang K. Hou C. Cai S. Huang Y.. (2016). Baicalein inhibits *Staphylococcus aureus* biofilm formation and the quorum sensing system *in vitro*. PLoS One 11:e0153468. doi: 10.1371/journal.pone.0153468, PMID: 27128436 PMC4851419

[ref57] Chen M. Yu Q. Sun H. (2013). Novel strategies for the prevention and treatment of biofilm related infections. Int. J. Mol. Sci. 14, 18488–18501. doi: 10.3390/ijms140918488, PMID: 24018891 PMC3794791

[ref58] Chidre P. Chavan A. Hulikunte Mallikarjunaiah N. Chandrakanth Revanasiddappa K. (2023). Nanomaterials: potential broad spectrum antimicrobial agents. Nat. Prod. Commun. 18:1934578X221106904. doi: 10.1177/1934578X221106904, PMID: 39711748

[ref59] Chopra H. Bibi S. Singh I. Hasan M. M. Khan M. S. Yousafi Q.. (2022). Green metallic nanoparticles: biosynthesis to applications. Front. Bioeng. Biotechnol. 10:874742. doi: 10.3389/fbioe.2022.874742, PMID: 35464722 PMC9019488

[ref60] Chouhan S. Sharma K. Guleria S. (2017). Antimicrobial activity of some essential oils-present status and future perspectives. Medicines 4:58. doi: 10.3390/medicines4030058, PMID: 28930272 PMC5622393

[ref61] Chuesiang P. Siripatrawan U. Sanguandeekul R. Yang J. S. McClements D. J. McLandsborough L. (2019). Antimicrobial activity and chemical stability of cinnamon oil in oil-in-water nanoemulsions fabricated using the phase inversion temperature method. LWT 110, 190–196. doi: 10.1016/j.lwt.2019.03.012

[ref62] Confessor M. V. A. Agreles M. A. A. Campos L. A. A. Silva Neto A. F. Borges J. C. Martins R. M.. (2024). Olive oil nanoemulsion containing curcumin: antimicrobial agent against multidrug-resistant bacteria. Appl. Microbiol. Biotechnol. 108:241. doi: 10.1007/s00253-024-13057-x, PMID: 38413482 PMC10899360

[ref63] Cui Y. Zhao Y. Tian Y. Zhang W. Lü X. Jiang X. (2011). The molecular mechanism of action of bactericidal gold nanoparticles on *Escherichia coli*. Biomaterials 33, 2327–2333. doi: 10.1016/j.biomaterials.2011.11.057, PMID: 22182745

[ref64] da Cunha K. F. Oliveira Garcia M. Allend S. O. de Albernaz D. F. T. Panagio L. A. Neto A. C. P. S.. (2023). Biogenic silver nanoparticles: *in vitro* activity against *Staphylococcus aureus* methicillin-resistant (MRSA) and multidrug-resistant coagulase-negative *Staphylococcus* (CoNS). Braz. J. Microbiol. 54, 2641–2650. doi: 10.1007/s42770-023-01102-2, PMID: 37676406 PMC10689704

[ref65] da Silva Gündel S. de Souza M. E. Quatrin P. M. Klein B. Wagner R. Gündel A.. (2018a). Nanoemulsions containing *Cymbopogon flexuosus* essential oil: development, characterization, stability study and evaluation of antimicrobial and antibiofilm activities. Microb. Pathog. 118, 268–276. doi: 10.1016/j.micpath.2018.03.043, PMID: 29581028

[ref66] da Silva Gündel S. Velho M. C. Diefenthaler M. K. Favarin F. R. Copetti P. M. de Oliveira Fogaça A.. (2018b). Basil oil-nanoemulsions: development, cytotoxicity and evaluation of antioxidant and antimicrobial potential. J. Drug Deliv. Sci. Technol. 46, 378–383. doi: 10.1016/j.jddst.2018.05.038

[ref67] Daniel M. Imtiaz-Umer S. Fergie N. Birchall J. P. Bayston R. (2012). Bacterial involvement in otitis media with effusion. Int. J. Pediatr. Otorhinolaryngol. 76, 1416–1422. doi: 10.1016/j.ijporl.2012.06.013, PMID: 22819485

[ref68] Das S. Singh V. K. Dwivedy A. K. Chaudhari A. K. Deepika Dubey N. K. (2021). Eugenol loaded chitosan nanoemulsion for food protection and inhibition of aflatoxin B1 synthesizing genes based on molecular docking. Carbohydr. Polym. 255:117339. doi: 10.1016/j.carbpol.2020.117339, PMID: 33436182

[ref69] Dasgupta A. (2020). “Chapter 19—toxic herbals and plants in the United States” in Toxicology cases for the clinical and forensic laboratory. eds. Ketha H. Garg U. (New York, NY: Academic Press), 359–368.

[ref70] Dash P. Rana K. Turuk J. Palo S. K. Pati S. (2023). Antimicrobial resistance and biofilm formation of *Staphylococcus aureus* isolates from febrile cases: findings from a rural cohort of Odisha, India. Pol. J. Microbiol. 72, 209–214. doi: 10.33073/pjm-2023-005, PMID: 37013928 PMC10266288

[ref71] De Maio F. Palmieri V. Salustri A. Perini G. Sanguinetti M. De Spirito M.. (2019). Graphene oxide prevents mycobacteria entry into macrophages through extracellular entrapment. Nanoscale Adv. 1, 1421–1431. doi: 10.1039/c8na00413g, PMID: 36132595 PMC9419007

[ref72] De Maio F. Palmieri V. Santarelli G. Perini G. Salustri A. Palucci I.. (2020). Graphene oxide-linezolid combination as potential new anti-tuberculosis treatment. Nanomaterials 10:1431. doi: 10.3390/nano10081431, PMID: 32707988 PMC7466666

[ref73] Debnath S. K. Srivastava R. (2021). Drug delivery with carbon-based nanomaterials as versatile nanocarriers: progress and prospects. Front. Nanotechnol. 3:644564. doi: 10.3389/fnano.2021.644564

[ref74] Dhanapal J. Balaraman Ravindrran M. Pradeep P. S. Seshadri S. (2014). Antibacterial activity of anthraquinone encapsulated chitosan/poly (lactic acid) nanoparticles. Int. J. Pharma Bio Sci. 5, 20–28.

[ref75] Diban F. Di Lodovico S. Di Fermo P. D’Ercole S. D’Arcangelo S. Di Giulio M.. (2023). Biofilms in chronic wound infections: innovative antimicrobial approaches using the *in vitro* Lubbock chronic wound biofilm model. Int. J. Mol. Sci. 24:1004. doi: 10.3390/ijms24021004, PMID: 36674518 PMC9862456

[ref76] Din F. U. Aman W. Ullah I. Qureshi O. S. Mustapha O. Shafique S.. (2017). Effective use of nanocarriers as drug delivery systems for the treatment of selected tumors. Int. J. Nanomedicine 12, 7291–7309. doi: 10.2147/IJN.S146315, PMID: 29042776 PMC5634382

[ref77] Doan Thi T. U. Nguyen T. T. Thi Y. D. Ta Thi K. H. Phan B. T. Pham K. N. (2020). Green synthesis of ZnO nanoparticles using orange fruit peel extract for antibacterial activities. RSC Adv. 10, 23899–23907. doi: 10.1039/D0RA04926C, PMID: 35517333 PMC9055061

[ref78] Doghish A. S. Shehabeldine A. M. El-Mahdy H. A. Hassanin M. M. H. Al-Askar A. A. Marey S. A.. (2023). Thymus vulgaris oil nanoemulsion: synthesis, characterization, antimicrobial and anticancer activities. Molecules 28:6910. doi: 10.3390/molecules28196910, PMID: 37836753 PMC10574288

[ref79] Donadu M. G. Ferrari M. Behzadi P. Trong Le N. Usai D. Fiamma M.. (2024). Multifactorial action of lavender and lavandin oils against filamentous fungi. Nat. Prod. Res., 1–9. doi: 10.1080/14786419.2024.2301741, PMID: 38293715

[ref80] Donlan R. M. (2001). Biofilms and device-associated infections. Emerg. Infect. Dis. 7, 277–281. doi: 10.3201/eid0702.010226, PMID: 11294723 PMC2631701

[ref81] Dorri K. Modaresi F. Shakibaie M. R. Moazamian E. (2022). Effect of gold nanoparticles on the expression of efflux pump mexA and mexB genes of *Pseudomonas aeruginosa* strains by quantitative real-time PCR. Pharmacia 69, 125–133. doi: 10.3897/pharmacia.69.e77608

[ref82] Dosler S. Hacioglu M. Yilmaz F. N. Oyardi O. (2020). Biofilm modelling on the contact lenses and comparison of the in vitro activities of multipurpose lens solutions and antibiotics. PeerJ 8:e9419. doi: 10.7717/peerj.9419, PMID: 32612893 PMC7320721

[ref83] Durmus N. G. Taylor E. N. Kummer K. M. Webster T. J. (2013). Enhanced efficacy of superparamagnetic iron oxide nanoparticles against antibiotic-resistant biofilms in the presence of metabolites. Adv. Mater. 25, 5706–5713. doi: 10.1002/adma.201302627, PMID: 23963848

[ref84] Edson J. A. Kwon Y. J. (2016). Design, challenge, and promise of stimuli-responsive nanoantibiotics. Nano Converg. 3:26. doi: 10.1186/s40580-016-0085-7, PMID: 28191436 PMC5271158

[ref85] El Masry M. Bhasme P. Mathew-Steiner S. S. Smith J. Smeenge T. Roy S.. (2023). Swine model of biofilm infection and invisible wounds. J. Vis. Exp. 196:e65301. doi: 10.3791/65301, PMID: 37395583 PMC10655070

[ref86] Elbhnsawi N. A. Elwakil B. H. Hassanin A. H. Shehata N. Elshewemi S. S. Hagar M.. (2023). Nano-chitosan/eucalyptus oil/cellulose acetate nanofibers: manufacturing, antibacterial and wound healing activities. Membranes 13:604. doi: 10.3390/membranes13060604, PMID: 37367808 PMC10301581

[ref87] Eleraky N. E. Allam A. Hassan S. B. Omar M. M. (2020). Nanomedicine fight against antibacterial resistance: an overview of the recent pharmaceutical innovations. Pharmaceutics 12:142. doi: 10.3390/pharmaceutics12020142, PMID: 32046289 PMC7076477

[ref88] Elkordy A. Haj-Ahmad R. Zaki R. (2021). An overview on natural product drug formulations from conventional medicines to nanomedicines: past, present and future. J. Drug Deliv. Sci. Technol. 63:102459. doi: 10.1016/j.jddst.2021.102459, PMID: 39717863

[ref89] El-Tarabily K. A. El-Saadony M. T. Alagawany M. Arif M. Batiha G. E. Khafaga A. F.. (2021). Using essential oils to overcome bacterial biofilm formation and their antimicrobial resistance. Saudi J. Biol. Sci. 28, 5145–5156. doi: 10.1016/j.sjbs.2021.05.033, PMID: 34466092 PMC8380992

[ref90] Erdem A. Metzler D. Cha D. K. Huang C. P. (2015). The short-term toxic effects of TiO_2_ nanoparticles toward bacteria through viability, cellular respiration, and lipid peroxidation. Environ. Sci. Pollut. Res. 22, 17917–17924. doi: 10.1007/s11356-015-5018-1, PMID: 26165996

[ref91] Esmaeillou M. Zarrini G. Ahangarzadeh Rezaee M. Shahbazi Mojarrad J. Bahadori A. (2017). Vancomycin capped with silver nanoparticles as an antibacterial agent against multi-drug resistance bacteria. Adv. Pharm Bull. 7, 479–483. doi: 10.15171/apb.2017.058, PMID: 29071232 PMC5651071

[ref92] Fadli M. Chevalier J. Saad A. Mezrioui N.-E. Hassani L. Pages J.-M. (2011). Essential oils from Moroccan plants as potential chemosensitisers restoring antibiotic activity in resistant gram-negative bacteria. Int. J. Antimicrob. Agents 38, 325–330. doi: 10.1016/j.ijantimicag.2011.05.005, PMID: 21752605

[ref93] Farrar A. J. Farrar F. C. (2020). Clinical aromatherapy. Nurs. Clin. North Am. 55, 489–504. doi: 10.1016/j.cnur.2020.06.015, PMID: 33131627 PMC7520654

[ref94] Fasting C. Schalley C. A. Weber M. Seitz O. Hecht S. Koksch B.. (2012). Multivalency as a chemical organization and action principle. Angew. Chem. Int. Ed. Engl. 51, 10472–10498. doi: 10.1002/anie.201201114, PMID: 22952048

[ref95] Fazly Bazzaz B. S. Khameneh B. Namazi N. Iranshahi M. Davoodi D. Golmohammadzadeh S. (2018). Solid lipid nanoparticles (SLN) carrying *Eugenia caryophyllata* essential oil: the novel nanoparticulate systems with broad-spectrum antimicrobial activity. Lett. Appl. Microbiol. 66, 506–513. doi: 10.1111/lam.12886, PMID: 29569372

[ref1002] Firisa S. G. Muleta G. G. Yimer A. A. (2022). Synthesis of Nickel Oxide Nanoparticles and Copper-Doped Nickel Oxide Nanocomposites Using Phytolacca dodecandra L’Herit Leaf Extract and Evaluation of Its Antioxidant and Photocatalytic Activities. ACS omega. 7, 44720–44732. doi: 10.1021/acsomega.2c04042, PMID: 36530241 PMC9753499

[ref96] Fisher L. E. Hook A. L. Ashraf W. Yousef A. Barrett D. A. Scurr D. J.. (2015). Biomaterial modification of urinary catheters with antimicrobials to give long-term broadspectrum antibiofilm activity. J. Control. Release 202, 57–64. doi: 10.1016/j.jconrel.2015.01.037, PMID: 25639970

[ref97] Gabrielyan L. Badalyan H. Gevorgyan V. Trchounian A. (2020). Comparable antibacterial effects and action mechanisms of silver and iron oxide nanoparticles on *Escherichia coli* and *Salmonella typhimurium*. Sci. Rep. 10:13145. doi: 10.1038/s41598-020-70211-x, PMID: 32753725 PMC7403320

[ref98] Galloway W. R. J. D. Hodgkinson J. T. Bowden S. D. Welch M. Spring D. R. (2010). Quorum sensing in gram-negative bacteria: small-molecule modulation of AHL and AI-2 quorum sensing pathways. Chem. Rev. 111, 28–67. doi: 10.1021/cr100109t, PMID: 21182299

[ref99] Gao W. Zhang L. (2021). Nanomaterials arising amid antibiotic resistance. Nat. Rev. Microbiol. 19, 5–6. doi: 10.1038/s41579-020-00469-5, PMID: 33024312 PMC7538279

[ref100] Gbejuade H. O. Lovering A. M. Webb J. C. (2014). The role of microbial biofilms in prosthetic joint infections. Acta Orthop. 86, 147–158. doi: 10.3109/17453674.2014.966290, PMID: 25238433 PMC4404764

[ref101] Gkartziou F. Giormezis N. Spiliopoulou I. Antimisiaris S. G. (2021). Nanobiosystems for antimicrobial drug-resistant infections. Nanomaterials 11:1075. doi: 10.3390/nano11051075, PMID: 33922004 PMC8143556

[ref102] Gold K. Slay B. Knackstedt M. Gaharwar A. (2018). Antimicrobial activity of metal and metal-oxide based nanoparticles. Adv. Ther. 1:1700033. doi: 10.1002/adtp.201700033

[ref103] Gominet M. Compain F. Beloin C. Lebeaux D. (2017). Central venous catheters and biofilms: where do we stand in 2017? APMIS 125, 365–375. doi: 10.1111/apm.12665, PMID: 28407421

[ref104] Goswami A. G. Basu S. Banerjee T. Shukla V. K. (2023). Biofilm and wound healing: from bench to bedside. Eur. J. Med. Res. 28:157. doi: 10.1186/s40001-023-01121-7, PMID: 37098583 PMC10127443

[ref105] Grgić J. Šelo G. Planinić M. Tišma M. Bucić-Kojić A. (2020). Role of the encapsulation in bioavailability of phenolic compounds. Antioxidants 9:923. doi: 10.3390/antiox9100923, PMID: 32993196 PMC7601682

[ref106] Gupta A. Saleh N. M. Das R. Landis R. F. Bigdeli A. Motamedchaboki K.. (2017). Synergistic antimicrobial therapy using nanoparticles and antibiotics for the treatment of multidrug-resistant bacterial infection. Nano Futures 1:015004. doi: 10.1088/2399-1984/aa69fb

[ref107] Hada A.-M. Potara M. Astilean S. Cordaro A. Neri G. Malanga M.. (2022). Linezolid nanoAntiobiotics and SERS-nanoTags based on polymeric cyclodextrin bimetallic core-shell nanoarchitectures. Carbohydr. Polym. 293:119736. doi: 10.1016/j.carbpol.2022.119736, PMID: 35798431

[ref108] Hammami I. Alabdallah N. M. Al Jomaa A. Kamoun M. (2021). Gold nanoparticles: synthesis properties and applications. J. King Saud Univ. Sci. 33:101560. doi: 10.1016/j.jksus.2021.101560

[ref109] Haque M. Sartelli M. McKimm J. Abu Bakar M. (2018). Health care-associated infections—an overview. Infect. Drug Resist. 11, 2321–2333. doi: 10.2147/IDR.S177247, PMID: 30532565 PMC6245375

[ref110] Harper R. A. Carpenter G. H. Proctor G. B. Harvey R. D. Gambogi R. J. Geonnotti A. R.. (2018). Diminishing biofilm resistance to antimicrobial nanomaterials through electrolyte screening of electrostatic interactions. Colloids Surf. B 173, 392–399. doi: 10.1016/j.colsurfb.2018.09.018, PMID: 30317126

[ref111] Hashemzadeh M. Dezfuli A. A. Z. Nashibi R. Jahangirimehr F. Akbarian Z. A. (2020). Study of biofilm formation, structure and antibiotic resistance in *Staphylococcus saprophyticus* strains causing urinary tract infection in women in Ahvaz, Iran. New Microbes New Infect. 39:100831. doi: 10.1016/j.nmni.2020.100831, PMID: 33489239 PMC7807165

[ref112] Hassen G. Belete G. Carrera K. G. Iriowen R. O. Araya H. Alemu T.. (2022). Clinical implications of herbal supplements in conventional medical practice: a US perspective. Cureus 14:e26893. doi: 10.7759/cureus.26893, PMID: 35978741 PMC9375827

[ref113] He J. Huang S. Sun X. Han L. Chang C. Zhang W.. (2019). Carvacrol loaded solid lipid nanoparticles of propylene glycol monopalmitate and glyceryl monostearate: preparation, characterization, and synergistic antimicrobial activity. Nanomaterials 9:1162. doi: 10.3390/nano9081162, PMID: 31416170 PMC6723752

[ref114] Hemaiswarya S. Kruthiventi A. K. Doble M. (2008). Synergism between natural products and antibiotics against infectious diseases. Phytomedicine 15, 639–652. doi: 10.1016/j.phymed.2008.06.008, PMID: 18599280

[ref115] Hetta H. F. Ramadan Y. N. Al-Harbi A. I. Ahmed E. A. Battah B. Abd Ellah N. H.. (2023). Nanotechnology as a promising approach to combat multidrug resistant bacteria: a comprehensive review and future perspectives. Biomedicines 11. doi: 10.3390/biomedicines11020413, PMID: 36830949 PMC9953167

[ref116] Høiby N. (2002). Understanding bacterial biofilms in patients with cystic fibrosis: current and innovative approaches to potential therapies. J. Cyst. Fibros. 1, 249–254. doi: 10.1016/S1569-1993(02)00104-2, PMID: 15463822

[ref117] Hou Y. Cheng B. Zhou M. Fang R. Jiang M. Hou W.. (2014). Searching for synergistic bronchodilators and novel therapeutic regimens for chronic lung diseases from a traditional Chinese medicine, Qingfei Xiaoyan Wan. PLoS One 9:e113104. doi: 10.1371/journal.pone.0113104, PMID: 25397687 PMC4232530

[ref118] Huang H. Wan P. Luo X. Lu Y. Li X. Xiong W.. (2023). Tigecycline resistance-associated mutations in the MepA efflux pump in *Staphylococcus aureus*. Microbiol Spectr 11:e0063423. doi: 10.1128/spectrum.00634-23, PMID: 37432114 PMC10434020

[ref119] Huh A. J. Kwon Y. J. (2011). “Nanoantibiotics”: a new paradigm for treating infectious diseases using nanomaterials in the antibiotics resistant era. J. Control. Release 156, 128–145. doi: 10.1016/j.jconrel.2011.07.002, PMID: 21763369

[ref120] Huo Y. Liu Y. Xia M. Du H. Lin Z. Li B.. (2022). Nanocellulose-based composite materials used in drug delivery systems. Polymers 14:2648. doi: 10.3390/polym14132648, PMID: 35808693 PMC9268916

[ref121] Hussain A. Shakeel F. Singh S. K. Alsarra I. A. Faruk A. Alanazi F. K.. (2019). Solidified SNEDDS for the oral delivery of rifampicin: evaluation, proof of concept, *in vivo* kinetics, and *in silico* GastroPlus^TM^ simulation. Int. J. Pharm. 566, 203–217. doi: 10.1016/j.ijpharm.2019.05.061, PMID: 31132448

[ref122] Huston M. DeBella M. DiBella M. Gupta A. (2021). Green synthesis of nanomaterials. Nanomaterials 11:2130. doi: 10.3390/nano11082130, PMID: 34443960 PMC8400177

[ref123] Illamola S. M. Amaeze O. U. Krepkova L. V. Birnbaum A. K. Karanam A. Job K. M.. (2020). Use of herbal medicine by pregnant women: what physicians need to know. Front. Pharmacol. 10:1483. doi: 10.3389/fphar.2019.01483, PMID: 31998122 PMC6962104

[ref124] Ingle A. P. Duran N. Rai M. (2013). Bioactivity, mechanism of action, and cytotoxicity of copper-based nanoparticles: a review. Appl. Microbiol. Biotechnol. 98, 1001–1009. doi: 10.1007/s00253-013-5422-8, PMID: 24305741

[ref125] Issakhanian L. Behzadi P. (2019). Antimicrobial agents and urinary tract infections. Curr. Pharm. Des. 25, 1409–1423. doi: 10.2174/1381612825999190619130216, PMID: 31218955

[ref126] Jadoun S. Arif R. Jangid N. Meena R. (2021). Green synthesis of nanoparticles using plant extracts: a review. Environ. Chem. Lett. 19, 355–374. doi: 10.1007/s10311-020-01074-x

[ref127] Jamil B. Abbasi R. Abbasi S. Imran M. Khan S. Ihsan A.. (2016). Encapsulation of cardamom essential oil in chitosan nano-composites: *in-vitro* efficacy on antibiotic resistant bacterial pathogens and cytotoxicity studies. Front. Microbiol. 7:1580. doi: 10.3389/fmicb.2016.01580, PMID: 27757108 PMC5048087

[ref128] Javed R. Zia M. Naz S. Aisida S. O. Ain N. Ain N. U.. (2020). Role of capping agents in the application of nanoparticles in biomedicine and environmental remediation: recent trends and future prospects. J. Nanobiotechnology 18:172. doi: 10.1186/s12951-020-00704-4, PMID: 33225973 PMC7682049

[ref129] Jelinkova P. Mazumdar A. Sur V. P. Kociova S. Dolezelikova K. Jimenez A. M. J.. (2019). Nanoparticle-drug conjugates treating bacterial infections. J. Control. Release 307, 166–185. doi: 10.1016/j.jconrel.2019.06.013, PMID: 31226356

[ref130] Jiang H. Li X. Xing Z. Niu Q. Xu J. (2023). Intracellular activity of poly (DL-lactide-co-glycolide) nanoparticles encapsulated with prothionamide, pyrazinamide, levofloxacin, linezolid, or ethambutol on multidrug-resistant *Mycobacterium tuberculosis*. Curr. Drug Deliv. 20, 306–316. doi: 10.2174/1567201819666220511120215, PMID: 35546770

[ref131] Jitareanu A. Trifan A. Vieriu M. Caba I.-C. Martu I. Agoroaei L. (2022). Current trends in toxicity assessment of herbal medicines: a narrative review. Processes 11:83. doi: 10.3390/pr11010083

[ref132] Johansen B. Duval R. Sergere J. (2020). Antimicrobial spectrum of Titroleane™:a new potent anti-infective agent. Antibiotics 9:391. doi: 10.3390/antibiotics9070391, PMID: 32650521 PMC7400619

[ref133] Joshi R. K. (2016). “A perspective on the Phytopharmaceuticals responsible for the therapeutic applications” in Recent advances in drug delivery technology (Hershey, PA: IGI Global), 229–262.

[ref134] Jouybari M. A. Ahanjan M. Mirzaei B. Goli H. R. (2021). Role of aminoglycoside-modifying enzymes and 16S rRNA methylase (ArmA) in resistance of *Acinetobacter baumannii* clinical isolates against aminoglycosides. Rev. Soc. Bras. Med. Trop. 54:e05992020. doi: 10.1590/0037-8682-0599-2020, PMID: 33533819 PMC7849326

[ref135] Junejo B. Eryilmaz M. Rizvanoglu S. S. Palabiyik I. M. Ghumro T. Mallah A.. (2023). Pharmacological assessment of Co_3_O_4_, CuO, NiO and ZnO nanoparticles via antibacterial, anti-biofilm and anti-quorum sensing activities. Water Sci. Technol. 87, 2840–2851. doi: 10.2166/wst.2023.150, PMID: 37318927

[ref136] Kalaiyan G. Suresh S. Thambidurai S. Prabu M. Kumar S. K. Nalenthiran P.. (2020). Green synthesis of hierarchical copper oxide microleaf bundles using *Hibiscus cannabinus* leaf extract for antibacterial application. J. Mol. Struct. 1217:128379. doi: 10.1016/j.molstruc.2020.128379

[ref137] Kamaruzzaman N. F. Tan L. P. Mat Yazid K. A. Saeed S. I. Hamdan R. H. Choong S. S.. (2018). Targeting the bacterial protective armour; challenges and novel strategies in the treatment of microbial biofilm. Materials 11:1705. doi: 10.3390/ma11091705, PMID: 30217006 PMC6164881

[ref138] Kapoor G. Saigal S. Elongavan A. (2017). Action and resistance mechanisms of antibiotics: a guide for clinicians. J. Anaesthesiol. Clin. Pharmacol. 33, 300–305. doi: 10.4103/joacp.JOACP_349_15, PMID: 29109626 PMC5672523

[ref139] Karampatakis T. Tsergouli K. Behzadi P. (2023). Carbapenem-resistant *Klebsiella pneumoniae*: virulence factors, molecular epidemiology and latest updates in treatment options. Antibiotics 12:234. doi: 10.3390/antibiotics12020234, PMID: 36830145 PMC9952820

[ref140] Karampatakis T. Tsergouli K. Behzadi P. (2024). Pan-genome plasticity and virulence factors: a natural treasure trove for *Acinetobacter baumannii*. Antibiotic 13:257. doi: 10.3390/antibiotics13030257, PMID: 38534692 PMC10967457

[ref141] Karigoudar R. M. Karigoudar M. H. Wavare S. M. Mangalgi S. S. (2019). Detection of biofilm among uropathogenic Escherichia coli and its correlation with antibiotic resistance pattern. J. Lab. Physicians 11, 17–22. doi: 10.4103/JLP.JLP_98_18, PMID: 30983797 PMC6437818

[ref142] Karmakar S. Mukherjee S. Joseph N. Priyadarshini A. Ravichandran V. Rajasekharan S.. (2023). “Biofilm formation in acute and chronic respiratory infections caused by nosocomial gram-negative bacteria” in Microbial biofilms (Cambridge, MA: Cambridge University Press), 391–413.

[ref143] Karnwal A. Kumar G. Pant G. Hossain K. Ahmad A. Alshammari M. B. (2023). Perspectives on usage of functional nanomaterials in antimicrobial therapy for antibiotic-resistant bacterial infections. ACS Omega 8, 13492–13508. doi: 10.1021/acsomega.3c00110, PMID: 37091369 PMC10116640

[ref144] Karthik P. Jose P. A. Chellakannu A. Gurusamy S. Ananthappan P. Karuppathevan R.. (2024). Green synthesis of MnO_2_ nanoparticles from *Psidium guajava* leaf extract: morphological characterization, photocatalytic and DNA/BSA interaction studies. Int. J. Biol. Macromol. 258:128869. doi: 10.1016/j.ijbiomac.2023.128869, PMID: 38114013

[ref145] Katongole P. Nalubega F. Florence N. C. Asiimwe B. Andia I. (2020). Biofilm formation, antimicrobial susceptibility and virulence genes of Uropathogenic *Escherichia coli* isolated from clinical isolates in Uganda. BMC Infect. Dis. 20:453. doi: 10.1186/s12879-020-05186-1, PMID: 32600258 PMC7325280

[ref146] Kaur T. Putatunda C. Vyas A. Kumar G. (2020). Zinc oxide nanoparticles inhibit bacterial biofilm formation via altering cell membrane permeability. Prep. Biochem. Biotechnol. 51, 309–319. doi: 10.1080/10826068.2020.1815057, PMID: 32921268

[ref147] Kelele K. Kahsay M. H. Aklilu M. (2019). Green synthesis of CuO nanoparticles using leaf extract of *Catha edulis* and its antibacterial activity. J. Pharm. Pharmacol. 7, 327–342. doi: 10.17265/2328-2150/2019.06.007

[ref148] Khan A. U. Maryam L. Zarrilli R. (2017). Structure, genetics and worldwide spread of New Delhi Metallo-β-lactamase (NDM): a threat to public health. BMC Microbiol. 17:101. doi: 10.1186/s12866-017-1012-8, PMID: 28449650 PMC5408368

[ref149] Khan I. Saeed K. Khan I. (2019). Nanoparticles: properties, applications and toxicities. Arab. J. Chem. 12, 908–931. doi: 10.1016/j.arabjc.2017.05.011

[ref150] Khan S. A. Shahid S. Lee C. S. (2020). Green synthesis of gold and silver nanoparticles using leaf extract of *Clerodendrum inerme*; characterization, antimicrobial, and antioxidant activities. Biomol. Ther. 10:835. doi: 10.3390/biom10060835, PMID: 32486004 PMC7356939

[ref151] Kim K. S. Lee D. Song C. G. Kang P. M. (2015). Reactive oxygen species-activated nanomaterials as theranostic agents. Nanomedicine 10, 2709–2723. doi: 10.2217/nnm.15.108, PMID: 26328770 PMC4612518

[ref152] Kirtane A. R. Verma M. Karandikar P. Furin J. Langer R. Traverso G. (2021). Nanotechnology approaches for global infectious diseases. Nat. Nanotechnol. 16, 369–384. doi: 10.1038/s41565-021-00866-8, PMID: 33753915

[ref153] Kohanski M. A. Dwyer D. J. Collins J. J. (2010). How antibiotics kill bacteria: from targets to networks. Nat. Rev. Microbiol. 8, 423–435. doi: 10.1038/nrmicro2333, PMID: 20440275 PMC2896384

[ref154] Korona-Glowniak I. Wisniewska A. Juda M. Kielbik K. Niedzielska G. Malm A. (2020). Bacterial aetiology of chronic otitis media with effusion in children—risk factors. J. Otolaryngol. Head Neck Surg. 49:24. doi: 10.1186/s40463-020-00418-5, PMID: 32349795 PMC7191732

[ref155] Krausz A. E. Adler B. L. Cabral V. Navati M. Doerner J. Charafeddine R. A.. (2015). Curcumin-encapsulated nanoparticles as innovative antimicrobial and wound healing agent. Nanomedicine 11, 195–206. doi: 10.1016/j.nano.2014.09.004, PMID: 25240595 PMC4461434

[ref156] Lammari N. Louaer O. Meniai A. H. Elaissari A. (2020). Encapsulation of essential oils via nanoprecipitation process: overview, progress, challenges and prospects. Pharmaceutics 12:431. doi: 10.3390/pharmaceutics12050431, PMID: 32392726 PMC7284627

[ref157] Lee J. Y. Garcia C. V. Shin G. H. Kim J. T. (2019). Antibacterial and antioxidant properties of hydroxypropyl methylcellulose-based active composite films incorporating oregano essential oil nanoemulsions. LWT 106, 164–171. doi: 10.1016/j.lwt.2019.02.061

[ref158] Lee N.-Y. Ko W.-C. Hsueh P.-R. (2019). Nanoparticles in the treatment of infections caused by multidrug-resistant organisms. Front. Pharmacol. 10:1153. doi: 10.3389/fphar.2019.01153, PMID: 31636564 PMC6787836

[ref159] Li M. Liu Y. Gong Y. Yan X. Wang L. Zheng W.. (2023). Recent advances in nanoantibiotics against multidrug-resistant bacteria. Nanoscale Adv. 5, 6278–6317. doi: 10.1039/d3na00530e, PMID: 38024316 PMC10662204

[ref160] Li M. Ma Z. Zhu Y. Xia H. Yao M. Chu X.. (2016a). Toward a molecular understanding of the antibacterial mechanism of copper-bearing titanium alloys against *Staphylococcus aureus*. Adv. Healthc. Mater. 5, 557–566. doi: 10.1002/adhm.201500712, PMID: 26692564 PMC4785048

[ref161] Li Y. Su T. Zhang Y. Huang X. Li J. Li C. (2015). Liposomal co-delivery of daptomycin and clarithromycin at an optimized ratio for treatment of methicillin-resistant *Staphylococcus aureus* infection. Drug Deliv. 22, 627–637. doi: 10.3109/10717544.2014.880756, PMID: 24471983

[ref162] Li P. Yin R. Cheng J. Lin J. (2023). Bacterial biofilm formation on biomaterials and approaches to its treatment and prevention. Int. J. Mol. Sci. 24:1680. doi: 10.3390/ijms241411680, PMID: 37511440 PMC10380251

[ref163] Li M. Zhu L. Liu B. Du L. Jia X. Han L.. (2016b). Tea tree oil nanoemulsions for inhalation therapies of bacterial and fungal pneumonia. Colloids Surf. B 141, 408–416. doi: 10.1016/j.colsurfb.2016.02.017, PMID: 26895502

[ref164] Liakos I. L. Iordache F. Carzino R. Scarpellini A. Oneto M. Bianchini P.. (2018). Cellulose acetate—essential oil nanocapsules with antimicrobial activity for biomedical applications. Colloids Surf. B 172, 471–479. doi: 10.1016/j.colsurfb.2018.08.069, PMID: 30199764

[ref165] Liao S. Zhang Y. Pan X. Zhu F. Jiang C. Liu Q.. (2019). Antibacterial activity and mechanism of silver nanoparticles against multidrug-resistant *Pseudomonas aeruginosa*. Int. J. Nanomedicine 14, 1469–1487. doi: 10.2147/IJN.S191340, PMID: 30880959 PMC6396885

[ref166] Lin C.-C. Phua D.-H. Deng J.-F. Yang C.-C. (2011). Aconitine intoxication mimicking acute myocardial infarction. Hum. Exp. Toxicol. 30, 782–785. doi: 10.1177/0960327110385960, PMID: 20937638

[ref167] Lin J. Tujios S. (2023). Hidden dangers: herbal and dietary supplement induced hepatotoxicity. Liver 3, 618–636. doi: 10.3390/livers3040041

[ref168] Liu P. Chen G. Zhang J. (2022). A review of liposomes as a drug delivery system: current status of approved products, regulatory environments, and future perspectives. Molecules 27:1372. doi: 10.3390/molecules27041372, PMID: 35209162 PMC8879473

[ref169] Liu R. Memarzadeh K. Chang B. Zhang Y. Ma Z. Allaker R. P.. (2016). Antibacterial effect of copper-bearing titanium alloy (Ti-Cu) against Streptococcus mutans and *Porphyromonas gingivalis*. Sci. Rep. 6:29985. doi: 10.1038/srep29985, PMID: 27457788 PMC4960589

[ref170] Long K. S. Vester B. (2011). Resistance to linezolid caused by modifications at its binding site on the ribosome. Antimicrob. Agents Chemother. 56, 603–612. doi: 10.1128/AAC.05702-11, PMID: 22143525 PMC3264260

[ref171] Lu M. Dai T. Murray C. K. Wu M. X. (2018). Bactericidal property of oregano oil against multidrug-resistant clinical isolates. Front. Microbiol. 9. doi: 10.3389/fmicb.2018.02329, PMID: 30344513 PMC6182053

[ref172] Lu H. Zhang X. Khan S. A. Li W. Wan L. (2021). Biogenic synthesis of MnO_2_ nanoparticles with leaf extract of *Viola betonicifolia* for enhanced antioxidant, antimicrobial, cytotoxic, and biocompatible applications. Front. Microbiol. 12:761084. doi: 10.3389/fmicb.2021.761084, PMID: 34790185 PMC8591690

[ref173] Luis A. I. S. Campos E. V. R. de Oliveira J. L. Guilger-Casagrande M. de Lima R. Castanha R. F.. (2020). Zein nanoparticles impregnated with eugenol and garlic essential oils for treating fish pathogens. ACS Omega 5, 15557–15566. doi: 10.1021/acsomega.0c01716, PMID: 32637831 PMC7331071

[ref174] Ma X. Liu H. Liu Z. Wang Y. Zhong Z. Peng G.. (2023). Trichosporon asahii *PLA2* gene enhances drug resistance to azoles by improving drug efflux and biofilm formation. Int. J. Mol. Sci. 24:8855. doi: 10.3390/ijms24108855, PMID: 37240199 PMC10219205

[ref175] Macias-Valcayo A. Aguilera-Correa J.-J. Broncano A. Parron R. Auñon A. Garcia-Cañete J.. (2022). Comparative *in vitro* study of biofilm formation and antimicrobial susceptibility in gram-negative Bacilli isolated from prosthetic joint infections. Microbiol. Spectr. 10:e0085122. doi: 10.1128/spectrum.00851-22, PMID: 35876589 PMC9430931

[ref176] Magryś A. Olender A. Tchórzewska D. (2021). Antibacterial properties of *Allium sativum* L. against the most emerging multidrug-resistant bacteria and its synergy with antibiotics. Arch. Microbiol. 203, 2257–2268. doi: 10.1007/s00203-021-02248-z, PMID: 33638666 PMC8205873

[ref177] Makabenta J. M. V. Nabawy A. Li C.-H. Schmidt-Malan S. Patel R. Rotello V. M. (2020). Nanomaterial-based therapeutics for antibiotic-resistant bacterial infections. Nat. Rev. Microbiol. 19, 23–36. doi: 10.1038/s41579-020-0420-1, PMID: 32814862 PMC8559572

[ref178] Malka E. Perelshtein I. Lipovsky A. Shalom Y. Naparstek L. Perkas N.. (2013). Eradication of multi-drug resistant bacteria by a novel Zn-doped CuO nanocomposite. Small 9, 4069–4076. doi: 10.1002/smll.201301081, PMID: 23813908

[ref179] Mamun M. M. Sorinolu A. J. Munir M. Vejerano E. P. (2021). Nanoantibiotics: functions and properties at the nanoscale to combat antibiotic resistance. Front. Chem. 9:687660. doi: 10.3389/fchem.2021.687660, PMID: 34055750 PMC8155581

[ref180] Mancuso G. Midiri A. Gerace E. Biondo C. (2021). Bacterial antibiotic resistance: the most critical pathogens. Pathogens 10:1310. doi: 10.3390/pathogens10101310, PMID: 34684258 PMC8541462

[ref181] Manke A. Wang L. Rojanasakul Y. (2013). Mechanisms of nanoparticle-induced oxidative stress and toxicity. Biomed. Res. Int. 2013:942916. doi: 10.1155/2013/942916, PMID: 24027766 PMC3762079

[ref182] Manquián-Cerda K. Cruces E. Angélica Rubio M. Reyes C. Arancibia-Miranda N. (2017). Preparation of nanoscale iron (oxide, oxyhydroxides and zero-valent) particles derived from blueberries: reactivity, characterization and removal mechanism of arsenate. Ecotoxicol. Environ. Saf. 145, 69–77. doi: 10.1016/j.ecoenv.2017.07.004, PMID: 28708983

[ref183] Mapara N. Sharma M. Shriram V. Bharadwaj R. Mohite K. C. Kumar V. (2015). Antimicrobial potentials of *Helicteres isora* silver nanoparticles against extensively drug-resistant (XDR) clinical isolates of *Pseudomonas aeruginosa*. Appl. Microbiol. Biotechnol. 99, 10655–10667. doi: 10.1007/s00253-015-6938-x, PMID: 26362684

[ref184] Maruthapandi M. Gupta A. Saravanan A. Jacobi G. Banin E. Luong J. H. T.. (2023). Ultrasonic-assisted synthesis of lignin-capped Cu_2_O nanocomposite with antibiofilm properties. Ultrason. Sonochem. 92:106241. doi: 10.1016/j.ultsonch.2022.106241, PMID: 36470127 PMC9722477

[ref185] Masri A. Anwar A. Khan N. A. Siddiqui R. (2019). The use of nanomedicine for targeted therapy against bacterial infections. Antibiotics 8:260. doi: 10.3390/antibiotics8040260, PMID: 31835647 PMC6963790

[ref186] Masum M. I. Siddiqa M. M. Ali K. A. Zhang Y. Abdallah Y. Ibrahim E.. (2019). Biogenic synthesis of silver nanoparticles using phyllanthus emblicafruit extract and its inhibitory action against the pathogen acidovorax oryzaestrain RS-2 of rice bacterial brown stripe. Front. Microbiol. 10:820. doi: 10.3389/fmicb.2019.00820, PMID: 31110495 PMC6501729

[ref187] Mehmood A. Javid S. Khan M. F. Ahmad K. S. Mustafa A. (2022). *In vitro* total phenolics, total flavonoids, antioxidant and antibacterial activities of selected medicinal plants using different solvent systems. BMC Chem. 16:64. doi: 10.1186/s13065-022-00858-2, PMID: 36030245 PMC9419333

[ref188] Melke P. Sahlin P. Levchenko A. Jönsson H. (2010). A cell-based model for quorum sensing in heterogeneous bacterial colonies. PLoS Comput. Biol. 6:e1000819. doi: 10.1371/journal.pcbi.1000819, PMID: 20585545 PMC2887461

[ref189] Merghni A. Lassoued M. A. Voahangy Rasoanirina B. N. Moumni S. Mastouri M. (2022). Characterization of turpentine nanoemulsion and assessment of its antibiofilm potential against methicillin-resistant *Staphylococcus aureus*. Microb. Pathog. 166:105530. doi: 10.1016/j.micpath.2022.105530, PMID: 35429586

[ref190] Michael C. A. Dominey-Howes D. Labbate M. (2014). The antimicrobial resistance crisis: causes, consequences, and management. Front. Public Health 2:145. doi: 10.3389/fpubh.2014.00145, PMID: 25279369 PMC4165128

[ref191] Michaelis C. Grohmann E. (2023). Horizontal gene transfer of antibiotic resistance genes in biofilms. Antibiotics 12:328. doi: 10.3390/antibiotics12020328, PMID: 36830238 PMC9952180

[ref192] Miller K. P. Wang L. Benicewicz B. C. Decho A. W. (2015). Inorganic nanoparticles engineered to attack bacteria. Chem. Soc. Rev. 44, 7787–7807. doi: 10.1039/C5CS00041F, PMID: 26190826

[ref193] Mir M. Permana A. D. Ahmed N. Khan G. M. Rehman A. U. Donnelly R. F. (2020). Enhancement in site-specific delivery of carvacrol for potential treatment of infected wounds using infection responsive nanoparticles loaded into dissolving microneedles: a proof of concept study. Eur. J. Pharm. Biopharm. 147, 57–68. doi: 10.1016/j.ejpb.2019.12.008, PMID: 31883906

[ref194] Mirzaei R. Mohammadzadeh R. Alikhani M. Y. Shokri Moghadam M. Karampoor S. Kazemi S.. (2020). The biofilm-associated bacterial infections unrelated to indwelling devices. IUBMB Life 72, 1271–1285. doi: 10.1002/iub.2266, PMID: 32150327

[ref195] Modi S. Inwati G. K. Gacem A. Saquib Abullais S. Prajapati R. Yadav V. K.. (2022). Nanostructured antibiotics and their emerging medicinal applications: an overview of nanoantibiotics. Antibiotics 11:708. doi: 10.3390/antibiotics11060708, PMID: 35740115 PMC9219893

[ref196] Mohammadinejat M. Sepahi A. A. Alipour E. (2023). Antibacterial and anti-biofilm activities of silver nano particles conjugated to chitosan against multi-drug resistant bacteria. Clin. Lab. 69, 60–67. doi: 10.7754/Clin.Lab.2022.220315, PMID: 36649516

[ref197] Mohammed M. A. Salim M. T. A. Anwer B. E. Aboshanab K. M. Aboulwafa M. M. (2021). Impact of target site mutations and plasmid associated resistance genes acquisition on resistance of *Acinetobacter baumannii* to fluoroquinolones. Sci. Rep. 11:20136. doi: 10.1038/s41598-021-99230-y, PMID: 34635692 PMC8505613

[ref198] Mohanta Y. K. Chakrabartty I. Mishra A. K. Chopra H. Mahanta S. Avula S. K.. (2023). Nanotechnology in combating biofilm: a smart and promising therapeutic strategy. Front. Microbiol. 13:1028086. doi: 10.3389/fmicb.2022.1028086, PMID: 36938129 PMC10020670

[ref199] Motakef-Kazemi N. Yaqoubi M. (2020). Green synthesis and characterization of bismuth oxide nanoparticle using *Mentha Pulegium* extract. Iran. J. Pharm. Res. 19, 70–79. doi: 10.22037/ijpr.2019.15578.13190, PMID: 33224212 PMC7667547

[ref200] Mubeen B. Ansar A. N. Rasool R. Ullah I. Imam S. S. Alshehri S.. (2021). Nanotechnology as a novel approach in combating microbes providing an alternative to antibiotics. Antibiotics 10:1473. doi: 10.3390/antibiotics10121473, PMID: 34943685 PMC8698349

[ref201] Munita J. M. Arias C. A. (2016). Mechanisms of antibiotic resistance. Microbiol. Spectr. 4. doi: 10.1128/microbiolspec.VMBF-0016-2015, PMID: 27227291 PMC4888801

[ref202] Naaz S. Shet V. Mujawar M. (2023). Green synthesis of copper oxide nanoparticles: characterization and applications for environmental and biomedical fields. Can. J. Chem. Eng. 102, 1454–1465. doi: 10.1002/cjce.25142, PMID: 39714112

[ref203] Nasim N. Sandeep I. S. Mohanty S. (2022). Plant-derived natural products for drug discovery: current approaches and prospects. Nucleus 65, 399–411. doi: 10.1007/s13237-022-00405-3, PMID: 36276225 PMC9579558

[ref204] Nasra S. Meghani N. Kumar A. (2023). Nanoemulsion-based system as a novel and promising approach for enhancing the antimicrobial and Antitumoral activity of *Thymus vulgaris* (L.) oil in human hepatocellular carcinoma cells. Appl. Biochem. Biotechnol. 196, 949–970. doi: 10.1007/s12010-023-04571-1, PMID: 37273096

[ref205] Naziri Z. Majlesi M. (2022). Comparison of the prevalence, antibiotic resistance patterns, and biofilm formation ability of methicillin-resistant *Staphylococcus pseudintermedius* in healthy dogs and dogs with skin infections. Vet. Res. Commun. 47, 713–721. doi: 10.1007/s11259-022-10032-7, PMID: 36327008

[ref206] Newman D. J. Cragg G. M. (2016). Natural products as sources of new drugs from 1981 to 2014. J. Nat. Prod. 79, 629–661. doi: 10.1021/acs.jnatprod.5b01055, PMID: 26852623

[ref207] Nguyen N. T. T. Nguyen L. M. Nguyen T. T. T. Nguyen T. T. Nguyen D. T. C. Van Tran T. (2022). Formation, antimicrobial activity, and biomedical performance of plant-based nanoparticles: a review. Environ. Chem. Lett. 20, 2531–2571. doi: 10.1007/s10311-022-01425-w, PMID: 35369682 PMC8956152

[ref208] Nikolic D. Bosco L. Moschetti M. Tinnirello V. Pucci M. Corleone V.. (2023). Anti-inflammatory properties of an aldehydes-enriched fraction of grapefruit essential oil. J. Food Sci. 88, 1172–1187. doi: 10.1111/1750-3841.16461, PMID: 36651875

[ref209] Nikolic P. Mudgil P. (2023). The cell wall, cell membrane and virulence factors of *Staphylococcus aureus* and their role in antibiotic resistance. Microorganisms 11:259. doi: 10.3390/microorganisms11020259, PMID: 36838224 PMC9965861

[ref210] Nishino K. Yamasaki S. Nakashima R. Zwama M. Hayashi-Nishino M. (2021). Function and inhibitory mechanisms of multidrug efflux pumps. Front. Microbiol. 12:737288. doi: 10.3389/fmicb.2021.737288, PMID: 34925258 PMC8678522

[ref211] Noor A. Jamil S. Sadeq T. Sheet M. Ameen M. Kohli K. (2023). Development and evaluation of nanoformulations containing timur oil and rosemary oil for treatment of topical fungal infections. Gels 9:516. doi: 10.3390/gels9070516, PMID: 37504395 PMC10378787

[ref212] Nzilu D. Madivoli E. Makhanu D. Wanakai S. Kiprono G. Kareru P. (2023). Green synthesis of copper oxide nanoparticles and its efficiency in degradation of rifampicin antibiotic. Sci. Rep. 13:14030. doi: 10.1038/s41598-023-41119-z, PMID: 37640783 PMC10462644

[ref213] Olesky M. Hobbs M. Nicholas R. A. (2002). Identification and analysis of amino acid mutations in porin IB that mediate intermediate-level resistance to penicillin and tetracycline in *Neisseria gonorrhoeae*. Antimicrob. Agents Chemother. 46, 2811–2820. doi: 10.1128/AAC.46.9.2811-2820.2002, PMID: 12183233 PMC127413

[ref214] Othman L. Sleiman A. Abdel-Massih R. M. (2019). Antimicrobial activity of polyphenols and alkaloids in middle eastern plants. Front. Microbiol. 10:911. doi: 10.3389/fmicb.2019.00911, PMID: 31156565 PMC6529554

[ref215] Ouerghemmi I. Rebey I. B. Rahali F. Z. Bourgou S. Pistelli L. Ksouri R.. (2016). Antioxidant and antimicrobial phenolic compounds from extracts of cultivated and wild-grown Tunisian *Ruta chalepensis*. J. Food Drug Anal. 25, 350–359. doi: 10.1016/j.jfda.2016.04.001, PMID: 28911677 PMC9332523

[ref216] Ozdal M. Gurkok S. (2022). Recent advances in nanoparticles as antibacterial agent. ADMET DMPK 10, 115–129. doi: 10.5599/admet.1172, PMID: 35350114 PMC8957245

[ref217] Ozkan G. Ceyhan T. Çatalkaya G. Rajan L. Ullah H. Daglia M.. (2024). Encapsulated phenolic compounds: clinical efficacy of a novel delivery method. Phytochem. Rev. 23, 781–819. doi: 10.1007/s11101-023-09909-5

[ref218] Pang Z. Raudonis R. Glick B. R. Lin T.-J. Cheng Z. (2018). Antibiotic resistance in *Pseudomonas aeruginosa*: mechanisms and alternative therapeutic strategies. Biotechnol. Adv. 37, 177–192. doi: 10.1016/j.biotechadv.2018.11.013, PMID: 30500353

[ref219] Parham S. Kharazi A. Z. Bakhsheshi-Rad H. R. Nur H. Ismail A. F. Sharif S.. (2020). Antioxidant, antimicrobial and antiviral properties of herbal materials. Antioxidants 9:1309. doi: 10.3390/antiox9121309, PMID: 33371338 PMC7767362

[ref220] Parra-Ortiz E. Caselli L. Agnoletti M. Skoda M. W. A. Li X. Zhao D.. (2022). Mesoporous silica as a matrix for photocatalytic titanium dioxide nanoparticles: lipid membrane interactions. Nanoscale 14, 12297–12312. doi: 10.1039/D2NR01958B, PMID: 35960150

[ref221] Patra J. K. Das G. Fraceto L. F. Campos E. V. R. del Pilar Rodriguez-Torres M. Acosta-Torres L. S.. (2018). Nano based drug delivery systems: recent developments and future prospects. J. Nanobiotechnology 16:71. doi: 10.1186/s12951-018-0392-8, PMID: 30231877 PMC6145203

[ref222] Pelling H. Nzakizwanayo J. Milo S. Denham E. L. MacFarlane W. M. Bock L. J.. (2019). Bacterial biofilm formation on indwelling urethral catheters. Lett. Appl. Microbiol. 68, 277–293. doi: 10.1111/lam.13144, PMID: 30811615

[ref223] Perez A. P. Perez N. Lozano C. M. S. Altube M. J. de Farias M. A. Portugal R. V.. (2019). The anti MRSA biofilm activity of *Thymus vulgaris* essential oil in nanovesicles. Phytomedicine 57, 339–351. doi: 10.1016/j.phymed.2018.12.025, PMID: 30826631

[ref224] Pérez-Conesa D. Cao J. Chen L. McLandsborough L. Weiss J. (2011). Inactivation of listeria monocytogenes and *Escherichia coli* O157:H7 biofilms by micelle-encapsulated eugenol and carvacrol. J. Food Prot. 74, 55–62. doi: 10.4315/0362-028X.JFP-08-403, PMID: 21219763

[ref225] Perveen K. Husain F. M. Qais F. A. Khan A. Razak S. Afsar T.. (2021). Microwave-assisted rapid green synthesis of gold nanoparticles using seed extract of *Trachyspermum ammi*: ROS mediated biofilm inhibition and anticancer activity. Biomolecules 11:197. doi: 10.3390/biom11020197, PMID: 33573343 PMC7911733

[ref226] Peterson E. Kaur P. (2018). Antibiotic resistance mechanisms in bacteria: relationships between resistance determinants of antibiotic producers, environmental bacteria, and clinical pathogens. Front. Microbiol. 9:2928. doi: 10.3389/fmicb.2018.02928, PMID: 30555448 PMC6283892

[ref227] Pfalzgraff A. Brandenburg K. Weindl G. (2018). Antimicrobial peptides and their therapeutic potential for bacterial skin infections and wounds. Front. Pharmacol. 9:281. doi: 10.3389/fphar.2018.00281, PMID: 29643807 PMC5882822

[ref228] Pichavant L. Carrié H. Nguyen M. N. Plawinski L. Durrieu M.-C. Héroguez V. (2016). Vancomycin functionalized nanoparticles for bactericidal biomaterial surfaces. Biomacromolecules 17, 1339–1346. doi: 10.1021/acs.biomac.5b01727, PMID: 26938371

[ref229] Posadzki P. Alotaibi A. Ernst E. (2012). Adverse effects of aromatherapy: a systematic review of case reports and case series. Int. J. Risk Saf. Med. 24, 147–161. doi: 10.3233/JRS-2012-0568, PMID: 22936057

[ref230] Pouget C. Dunyach-Remy C. Magnan C. Pantel A. Sotto A. Lavigne J.-P. (2022). Polymicrobial biofilm organization of *Staphylococcus aureus* and *Pseudomonas aeruginosa* in a chronic wound environment. Int. J. Mol. Sci. 23:761. doi: 10.3390/ijms231810761, PMID: 36142675 PMC9504628

[ref231] Prakash J. Cho J. Mishra Y. K. (2022). Photocatalytic TiO_2_ nanomaterials as potential antimicrobial and antiviral agents: scope against blocking the SARS-COV-2 spread. Micro Nano Eng. 14:100100. doi: 10.1016/j.mne.2021.100100 PMC8685168 40477493

[ref232] Preda V. G. Săndulescu O. (2019). Communication is the key: biofilms, quorum sensing, formation and prevention. Discoveries 7:e100:e10. doi: 10.15190/d.2019.13, PMID: 32309618 PMC7086079

[ref233] Prestinaci F. Pezzotti P. Pantosti A. (2015). Antimicrobial resistance: a global multifaceted phenomenon. Pathog. Glob. Health 109, 309–318. doi: 10.1179/2047773215Y.0000000030, PMID: 26343252 PMC4768623

[ref234] Qin S. Xiao W. Zhou C. Pu Q. Deng X. Lan L.. (2022). *Pseudomonas aeruginosa*: pathogenesis, virulence factors, antibiotic resistance, interaction with host, technology advances and emerging therapeutics. Signal Transduct. Target. Ther. 7:199. doi: 10.1038/s41392-022-01056-1, PMID: 35752612 PMC9233671

[ref235] Quatrin P. M. Verdi C. M. de Souza M. E. de Godoi S. N. Klein B. Gundel A.. (2017). Antimicrobial and antibiofilm activities of nanoemulsions containing *Eucalyptus globulus* oil against *Pseudomonas aeruginosa* and *Candida* spp. Microb. Pathog. 112, 230–242. doi: 10.1016/j.micpath.2017.09.062, PMID: 28970174

[ref236] Rahman F. Patwary M. A. Siddique M. Bashar M. Haque M. Akter B.. (2022). Green synthesis of zinc oxide nanoparticles using *Cocos nucifera* leaf extract: characterization, antimicrobial, antioxidant and photocatalytic activity. R. Soc. Open Sci. 9:220858. doi: 10.1098/rsos.220858, PMID: 36425517 PMC9682308

[ref237] Raksha L. Gangashettappa N. Shantala G. B. Nandan B. R. Sinha D. (2020). Study of biofilm formation in bacterial isolates from contact lens wearers. Indian J. Ophthalmol. 68, 23–28. doi: 10.4103/ijo.IJO_947_19, PMID: 31856459 PMC6951123

[ref238] Ramadan R. Omar N. Dawaba M. Moemen D. (2021). Bacterial biofilm dependent catheter associated urinary tract infections: characterization, antibiotic resistance pattern and risk factors. Egypt. J. Basic Appl. Sci. 8, 64–74. doi: 10.1080/2314808X.2021.1905464

[ref239] Ramírez Castillo F. Y. Guerrero Barrera A. L. Harel J. Avelar González F. J. Vogeleer P. Arreola Guerra J. M.. (2023). Biofilm formation by *Escherichia coli* isolated from urinary tract infections from Aguascalientes, Mexico. Microorganisms 11:2858. doi: 10.3390/microorganisms11122858, PMID: 38138002 PMC10745304

[ref240] Rana R. Awasthi R. Sharma B. Kulkarni G. T. (2019). Nanoantibiotic formulations to combat antibiotic resistance-old wine in a new bottle. Recent Pat. Drug Deliv. Formul. 13, 174–183. doi: 10.2174/1872211313666190911124626, PMID: 31544718

[ref241] Rao S. Xu G. Zeng H. Zheng X. Hu Q. Wang Q.. (2020). Physicochemical and antibacterial properties of fabricated ovalbumin-carvacrol gel nanoparticles. Food Funct. 11, 5133–5141. doi: 10.1039/D0FO00755B, PMID: 32432306

[ref242] Rashidipour M. Ashrafi B. Nikbakht M. R. Veiskarami S. Taherikalani M. Soroush S. (2021). Encapsulation of *Satureja khuzistanica* jamzad essential oil in chitosan nanoparticles with enhanced antibacterial and anticancer activities. Prep. Biochem. Biotechnol. 51, 971–978. doi: 10.1080/10826068.2021.1881907, PMID: 33586597

[ref243] Robino L. Scavone P. (2020). Nanotechnology in biofilm prevention. Future Microbiol. 15, 377–379. doi: 10.2217/fmb-2019-0327, PMID: 32242746

[ref244] Rochford E. T. J. Richards R. G. Moriarty T. F. (2012). Influence of material on the development of device-associated infections. Clin. Microbiol. Infect. 18, 1162–1167. doi: 10.1111/j.1469-0691.2012.04002.x, PMID: 22925523

[ref245] Rodenak-Kladniew B. Scioli Montoto S. Sbaraglini M. L. Di Ianni M. Ruiz M. E. Talevi A.. (2019). Hybrid ofloxacin/eugenol co-loaded solid lipid nanoparticles with enhanced and targetable antimicrobial properties. Int. J. Pharm. 569:118575. doi: 10.1016/j.ijpharm.2019.118575, PMID: 31356956

[ref246] Rostock L. Driller R. Grätz S. Kerwat D. Eckardstein L. Petras D.. (2018). Molecular insights into antibiotic resistance—how a binding protein traps albicidin. Nat. Commun. 9:3095. doi: 10.1038/s41467-018-05551-4, PMID: 30082794 PMC6078987

[ref247] Roy S. Santra S. Das A. Dixith S. Sinha M. Ghatak S.. (2020). *Staphylococcus aureus* biofilm infection compromises wound healing by causing deficiencies in granulation tissue collagen. Ann. Surg. 271, 1174–1185. doi: 10.1097/SLA.0000000000003053, PMID: 30614873 PMC7065840

[ref248] Rudolf J. D. Bigelow L. Chang C. Cuff M. E. Lohman J. R. Chang C.-Y.. (2015). Crystal structure of the zorbamycin-binding protein ZbmA, the primary self-resistance element in *Streptomyces flavoviridis* ATCC21892. Biochemistry 54, 6842–6851. doi: 10.1021/acs.biochem.5b01008, PMID: 26512730 PMC4809751

[ref249] Sadovskaya I. Chaignon P. Kogan G. Chokr A. Vinogradov E. Jabbouri S. (2006). Carbohydrate-containing components of biofilms produced in vitro by some staphylococcal strains related to orthopaedic prosthesis infections. FEMS Immunol. Med. Microbiol. 47, 75–82. doi: 10.1111/j.1574-695X.2006.00068.x, PMID: 16706790

[ref250] Saha B. Bhattacharya J. Mukherjee A. Ghosh A. K. Santra C. R. Dasgupta A. K.. (2007). *In vitro* structural and functional evaluation of gold nanoparticles conjugated antibiotics. Nanoscale Res. Lett. 2, 614–622. doi: 10.1007/s11671-007-9104-2

[ref251] Saha M. Sarkar A. (2021). Review on multiple facets of drug resistance: a rising challenge in the 21st century. J. Xenobiot. 11, 197–214. doi: 10.3390/jox11040013, PMID: 34940513 PMC8708150

[ref252] Salam M. A. Al-Amin M. Y. Salam M. T. Pawar J. S. Akhter N. Rabaan A. A.. (2023). Antimicrobial resistance: a growing serious threat for global public health. Healthcare 11:1946. doi: 10.3390/healthcare11131946, PMID: 37444780 PMC10340576

[ref253] Salustri A. De Maio F. Palmieri V. Santarelli G. Palucci I. Bianco D.. (2023). Evaluation of the toxic activity of the graphene oxide in the *ex vivo* model of human PBMC infection with *Mycobacterium tuberculosis*. Microorganisms 11:554. doi: 10.3390/microorganisms11030554, PMID: 36985128 PMC10059016

[ref254] Savoia D. (2012). Plant-derived antimicrobial compounds: alternatives to antibiotics. Future Microbiol. 7, 979–990. doi: 10.2217/fmb.12.68, PMID: 22913356

[ref255] Saxena S. Punjabi K. Ahamad N. Singh S. Bendale P. Banerjee R. (2022). Nanotechnology approaches for rapid detection and theranostics of antimicrobial resistant bacterial infections. ACS Biomater Sci. Eng. 8, 2232–2257. doi: 10.1021/acsbiomaterials.1c01516, PMID: 35546526

[ref256] Schilcher K. Horswill A. R. (2020). Staphylococcal biofilm development: structure, regulation, and treatment strategies. Microbiol. Mol. Biol. Rev. 84:e00026. doi: 10.1128/MMBR.00026-19, PMID: 32792334 PMC7430342

[ref257] Seyyedi-Mansour S. Rodríguez M. Echave J. Rivo F. N. González Pereira A. Soria López A.. (2022). Plant-based nanoantibiotics: an effective strategy to overcoming on antibiotic resistance. Med. Sci. Forum 12:12. doi: 10.3390/eca2022-12727

[ref258] Shaikh S. Nazam N. Rizvi S. M. D. Ahmad K. Baig M. H. Lee E. J.. (2019). Mechanistic insights into the antimicrobial actions of metallic nanoparticles and their implications for multidrug resistance. Int. J. Mol. Sci. 20:2468. doi: 10.3390/ijms20102468, PMID: 31109079 PMC6566786

[ref259] Sharifi-Rad J. Sureda A. Tenore G. C. Daglia M. Sharifi-Rad M. Valussi M.. (2017). Biological activities of essential oils: from plant chemoecology to traditional healing systems. Molecules 22:70. doi: 10.3390/molecules22010070, PMID: 28045446 PMC6155610

[ref260] Shree P. Singh C. K. Sodhi K. K. Surya J. N. Singh D. K. (2023). Biofilms: understanding the structure and contribution towards bacterial resistance in antibiotics. Med. Microecol. 16:100084. doi: 10.1016/j.medmic.2023.100084

[ref261] Silva K. P. T. Sundar G. Khare A. (2023). Efflux pump gene amplifications bypass necessity of multiple target mutations for resistance against dual-targeting antibiotic. Nat. Commun. 14:3402. doi: 10.1038/s41467-023-38507-4, PMID: 37296157 PMC10256781

[ref262] Singh B. Basniwal R. Buttar H. S. Jain V. Jain N. (2011). Curcumin nanoparticles: preparation, characterization, and antimicrobial study. J. Agric. Food Chem. 59, 2056–2061. doi: 10.1021/jf104402t, PMID: 21322563

[ref263] Singh H. Desimone M. F. Pandya S. Jasani S. George N. Adnan M.. (2023). Revisiting the green synthesis of nanoparticles: uncovering influences of plant extracts as reducing agents for enhanced synthesis efficiency and its biomedical applications. Int. J. Nanomedicine 18, 4727–4750. doi: 10.2147/IJN.S419369, PMID: 37621852 PMC10444627

[ref264] Singh P. Garg A. Pandit S. Mokkapati V. R. S. S. Mijakovic I. (2018). Antimicrobial effects of biogenic nanoparticles. Nanomaterials 8:1009. doi: 10.3390/nano8121009, PMID: 30563095 PMC6315689

[ref265] Singh A. K. Kim J. Y. Lee Y. S. (2022). Phenolic compounds in active packaging and edible films/coatings: natural bioactive molecules and novel packaging ingredients. Molecules 27:7513. doi: 10.3390/molecules27217513, PMID: 36364340 PMC9655785

[ref266] Singh K. Panghal M. Kadyan S. Chaudhary U. Yadav J. P. (2014). Green silver nanoparticles of *Phyllanthus amarus*: as an antibacterial agent against multi drug resistant clinical isolates of *Pseudomonas aeruginosa*. J. Nanobiotechnology 12:40. doi: 10.1186/s12951-014-0040-x, PMID: 25271044 PMC4189661

[ref267] Singh A. Zhao K. (2017). “Chapter nine-herb-drug interactions of commonly used Chinese medicinal herbs” in International review of neurobiology. eds. Zeng B.-Y. Zhao K. (New York: Academic Press), 197–232. 10.1016/bs.irn.2017.02.010 28807159

[ref268] Sirelkhatim A. Mahmud S. Seeni A. Kaus N. H. M. Ann L. C. Bakhori S. K. M.. (2015). Review on zinc oxide nanoparticles: antibacterial activity and toxicity mechanism. Nanomicro Lett. 7, 219–242. doi: 10.1007/s40820-015-0040-x, PMID: 30464967 PMC6223899

[ref269] Soares S. Sousa J. Pais A. Vitorino C. (2018). Nanomedicine: principles, properties, and regulatory issues. Front. Chem. 6:360. doi: 10.3389/fchem.2018.00360, PMID: 30177965 PMC6109690

[ref270] Sondi I. Salopek-Sondi B. (2004). Silver nanoparticles as antimicrobial agent: a case study on *E. coli* as a model for gram-negative bacteria. J. Colloid Interface Sci. 275, 177–182. doi: 10.1016/j.jcis.2004.02.012, PMID: 15158396

[ref271] Soren O. Rineh A. Silva D. G. Cai Y. Howlin R. P. Allan R. N.. (2020). Cephalosporin nitric oxide-donor prodrug DEA-C3D disperses biofilms formed by clinical cystic fibrosis isolates of *Pseudomonas aeruginosa*. J. Antimicrob. Chemother. 75, 117–125. doi: 10.1093/jac/dkz378, PMID: 31682251 PMC6910178

[ref272] Steindler L. Venturi V. (2007). Detection of quorum-sensing N-acyl homoserine lactone signal molecules by bacterial biosensors. FEMS Microbiol. Lett. 266, 1–9. doi: 10.1111/j.1574-6968.2006.00501.x, PMID: 17233715

[ref273] Stokes J. M. French S. Ovchinnikova O. G. Bouwman C. Whitfield C. Brown E. D. (2016). Cold stress makes *Escherichia coli* susceptible to glycopeptide antibiotics by altering outer membrane integrity. Cell Chem. Biol. 23, 267–277. doi: 10.1016/j.chembiol.2015.12.011, PMID: 26853624

[ref274] Sugumar S. Mukherjee A. Chandrasekaran N. (2015). Eucalyptus oil nanoemulsion-impregnated chitosan film: antibacterial effects against a clinical pathogen, *Staphylococcus aureus*, *in vitro*. Int. J. Nanomedicine 10, 67–75. doi: 10.2147/IJN.S79982, PMID: 26491308 PMC4599619

[ref275] Sultana A. Zare M. Thomas V. Kumar T. S. S. Ramakrishna S. (2022). Nano-based drug delivery systems: conventional drug delivery routes, recent developments and future prospects. Med. Drug Discov. 15:100134. doi: 10.1016/j.medidd.2022.100134

[ref276] Sundararajan B. Moola A. K. Vivek K. Kumari B. D. R. (2018). Formulation of nanoemulsion from leaves essential oil of *Ocimum basilicum* L. and its antibacterial, antioxidant and larvicidal activities (*Culex quinquefasciatus*). Microb. Pathog. 125, 475–485. doi: 10.1016/j.micpath.2018.10.017, PMID: 30340015

[ref277] Surette M. D. Spanogiannopoulos P. Wright G. D. (2021). The enzymes of the rifamycin antibiotic resistome. Acc. Chem. Res. 54, 2065–2075. doi: 10.1021/acs.accounts.1c00048, PMID: 33877820

[ref278] Svensson Malchau K. Tillander J. Zaborowska M. Hoffman M. Lasa I. Thomsen P.. (2021). Biofilm properties in relation to treatment outcome in patients with first-time periprosthetic hip or knee joint infection. J. Orthop. Translat. 30, 31–40. doi: 10.1016/j.jot.2021.05.008, PMID: 34485075 PMC8385121

[ref279] Tang J. Ouyang Q. Li Y. Zhang P. Jin W. Qu S.. (2022). Nanomaterials for delivering antibiotics in the therapy of pneumonia. Int. J. Mol. Sci. 23:5738. doi: 10.3390/ijms232415738, PMID: 36555379 PMC9779065

[ref280] Tariq F. N. Shafiq M. Khawar N. Habib G. Gul H. Hayat A.. (2023). The functional repertoire of AmpR in the AmpC β-lactamase high expression and decreasing β-lactam and aminoglycosides resistance in ESBL *Citrobacter freundii*. Heliyon 9:e19486. doi: 10.1016/j.heliyon.2023.e19486, PMID: 37662790 PMC10472055

[ref281] Teow S.-Y. Wong M. M.-T. Yap H.-Y. Peh S.-C. Shameli K. (2018). Bactericidal properties of plants-derived metal and metal oxide nanoparticles (NPs). Molecules 23:1366. doi: 10.3390/molecules23061366, PMID: 29882775 PMC6100366

[ref282] Tilahun E. Adimasu Y. Dessie Y. (2023). Biosynthesis and optimization of ZnO nanoparticles using Ocimum lamifolium leaf extract for electrochemical sensor and antibacterial activity. ACS Omega 8, 27344–27354. doi: 10.1021/acsomega.3c02709, PMID: 37546677 PMC10399153

[ref283] Tsikourkitoudi V. Henriques-Normark B. Sotiriou G. A. (2022). Inorganic nanoparticle engineering against bacterial infections. Curr. Opin. Chem. Eng. 38:100872. doi: 10.1016/j.coche.2022.100872

[ref284] Tungmunnithum D. Thongboonyou A. Pholboon A. Yangsabai A. (2018). Flavonoids and other phenolic compounds from medicinal plants for pharmaceutical and medical aspects: an overview. Medicines 5:93. doi: 10.3390/medicines5030093, PMID: 30149600 PMC6165118

[ref285] Uddin T. M. Chakraborty A. J. Khusro A. Zidan B. R. M. Mitra S. Emran T. B.. (2021). Antibiotic resistance in microbes: history, mechanisms, therapeutic strategies and future prospects. J. Infect. Public Health 14, 1750–1766. doi: 10.1016/j.jiph.2021.10.020, PMID: 34756812

[ref286] Ullah S. Khan M. N. (2022). Classification, synthetic, and characterization approaches to nanoparticles, and their applications in various fields of nanotechnology: a review. Catalysts 12:1386. doi: 10.3390/catal12111386, PMID: 39659294

[ref287] Vallet-Regí M. Balas F. Arcos D. (2007). Mesoporous materials for drug delivery. Angew. Chem. Int. Ed. 46, 7548–7558. doi: 10.1002/anie.200604488, PMID: 17854012

[ref288] Van Hoecke H. De Paepe A.-S. Lambert E. Van Belleghem J. D. Cools P. Van Simaey L.. (2016). *Haemophilus influenzae* biofilm formation in chronic otitis media with effusion. Eur. Arch. Otorrinolaringol. 273, 3553–3560. doi: 10.1007/s00405-016-3958-9, PMID: 26946303

[ref289] Varma A. Warghane A. Dhiman N. K. Paserkar N. Upadhye V. Modi A.. (2023). The role of nanocomposites against biofilm infections in humans. Front. Cell. Infect. Microbiol. 13:1104615. doi: 10.3389/fcimb.2023.1104615, PMID: 36926513 PMC10011468

[ref290] Vassallo A. Silletti M. F. Faraone I. Milella L. (2020). Nanoparticulate antibiotic systems as antibacterial agents and antibiotic delivery platforms to fight infections. J. Nanomater. 2020, 1–31. doi: 10.1155/2020/6905631, PMID: 39136038

[ref291] von Wintersdorff C. J. H. Penders J. van Niekerk J. M. Mills N. D. Majumder S. van Alphen L. B.. (2016). Dissemination of antimicrobial resistance in microbial ecosystems through horizontal gene transfer. Front. Microbiol. 7:173. doi: 10.3389/fmicb.2016.00173, PMID: 26925045 PMC4759269

[ref292] Wahab S. Khan T. Adil M. Khan A. (2021). Mechanistic aspects of plant-based silver nanoparticles against multi-drug resistant bacteria. Heliyon 7:e07448. doi: 10.1016/j.heliyon.2021.e07448, PMID: 34286126 PMC8273360

[ref293] Walsh C. (2000). Molecular mechanisms that confer antibacterial drug resistance. Nature 406, 775–781. doi: 10.1038/35021219, PMID: 10963607

[ref294] Wang K. Deng Y. Cui X. Chen M. Ou Y. Li D.. (2023). PatA regulates isoniazid resistance by mediating mycolic acid synthesis and controls biofilm formation by affecting lipid synthesis in mycobacteria. Microbiol. Spectr. 11:e0092823. doi: 10.1128/spectrum.00928-23, PMID: 37212713 PMC10269662

[ref295] Wang Z. Wen Z. Jiang M. Xia F. Wang M. Zhuge X.. (2022). Dissemination of virulence and resistance genes among *Klebsiella pneumoniae* via outer membrane vesicle: an important plasmid transfer mechanism to promote the emergence of carbapenem-resistant hypervirulent *Klebsiella pneumoniae*. Transbound. Emerg. Dis. 69, e2661–e2676. doi: 10.1111/tbed.14615, PMID: 35679514

[ref296] Wang S. Yu S. Lin Y. Zou P. Chai G. Yu H. H.. (2018). Co-delivery of ciprofloxacin and Colistin in liposomal formulations with enhanced *in vitro* antimicrobial activities against multidrug resistant *Pseudomonas aeruginosa*. Pharm. Res. 35:187. doi: 10.1007/s11095-018-2464-8, PMID: 30094660 PMC6122860

[ref297] Waters C. M. Bassler B. L. (2005). Quorum sensing: cell-to-cell communication in bacteria. Annu. Rev. Cell Dev. Biol. 21, 319–346. doi: 10.1146/annurev.cellbio.21.012704.131001, PMID: 16212498

[ref298] Weinberger M. Bronsky E. Bensch G. W. Bock G. N. Yecies J. J. (1975). Interaction of ephedrine and theophylline. Clin. Pharmacol. Ther. 17, 585–592. doi: 10.1002/cpt1975175585, PMID: 1092514

[ref299] Weisblum B. (1995). Erythromycin resistance by ribosome modification. Antimicrob. Agents Chemother. 39, 577–585. doi: 10.1128/AAC.39.3.577, PMID: 7793855 PMC162587

[ref300] Whittle E. E. McNeil H. E. Trampari E. Webber M. Overton T. W. Blair J. M. A. (2021). Efflux impacts intracellular accumulation only in actively growing bacterial cells. MBio 12:e0260821. doi: 10.1128/mBio.02608-21, PMID: 34634938 PMC8510537

[ref301] Wilks S. A. Koerfer V. V. Prieto J. A. Fader M. Keevil C. W. (2021). Biofilm development on urinary catheters promotes the appearance of viable but nonculturable bacteria. mBio 12:e03584. doi: 10.1128/mBio.03584-20, PMID: 33758085 PMC8092313

[ref302] Wink M. (2015). Modes of action of herbal medicines and plant secondary metabolites. Medicines 2, 251–286. doi: 10.3390/medicines2030251, PMID: 28930211 PMC5456217

[ref303] Wolcott R. (2021). Biofilm and catheter-related bloodstream infections. Br. J. Nurs. 30, S4–S9. doi: 10.12968/bjon.2021.30.8.S4, PMID: 33876689

[ref304] World Health Organization (2022). Global antimicrobial resistance and use surveillance system (GLASS) report 2022. Geneva: World Health Organization.

[ref305] Wu Z. L. Zhao J. Xu R. (2020). Recent advances in oral nano-antibiotics for bacterial infection therapy. Int. J. Nanomedicine 15, 9587–9610. doi: 10.2147/IJN.S279652, PMID: 33293809 PMC7719120

[ref306] Xavier B. B. Coppens J. de Koster S. Rajakani S. G. van Goethem S. Mzougui S.. (2021). Novel vancomycin resistance gene cluster in *Enterococcus faecium* ST1486, Belgium, June 2021. Eur. Secur. 26:2100767. doi: 10.2807/1560-7917.ES.2021.26.36.2100767, PMID: 34505571 PMC8431993

[ref307] Yadeta Gemachu L. Lealem Birhanu A. (2024). Green synthesis of ZnO, CuO and NiO nanoparticles using neem leaf extract and comparing their photocatalytic activity under solar irradiation. Green Chem. Lett. Rev. 17:2293841. doi: 10.1080/17518253.2023.2293841

[ref308] Yaghubi Kalurazi T. Jafari A. (2020). Evaluation of magnesium oxide and zinc oxide nanoparticles against multi-drug-resistance *Mycobacterium tuberculosis*. Indian J. Tuberc. 68, 195–200. doi: 10.1016/j.ijtb.2020.07.032, PMID: 33845951

[ref309] Yang B. Xie Y. Guo M. Rosner M. Yang H. Ronco C. (2018). Nephrotoxicity and Chinese herbal medicine. Clin. J. Am. Soc. Nephrol. 13, 1605–1611. doi: 10.2215/CJN.11571017, PMID: 29615394 PMC6218812

[ref310] Yasin A. Fatima U. Shahid S. Mansoor S. Inam H. Javed M.. (2022). Fabrication of copper oxide nanoparticles using *Passiflora edulis* extract for the estimation of antioxidant potential and photocatalytic methylene blue dye degradation. Agronomy 12:2315. doi: 10.3390/agronomy12102315

[ref311] Yeh Y.-C. Huang T.-H. Yang S.-C. Chen C.-C. Fang J.-Y. (2020). Nano-based drug delivery or targeting to eradicate bacteria for infection mitigation: a review of recent advances. Front. Chem. 8:286. doi: 10.3389/fchem.2020.00286, PMID: 32391321 PMC7193053

[ref312] Yi J. Sun Y. Zeng C. Kostoulias X. Qu Y. (2023). The role of biofilms in contact lens associated fungal keratitis. Antibiotics 12:1533. doi: 10.3390/antibiotics12101533, PMID: 37887234 PMC10604847

[ref313] Yoo H.-J. Yoon H.-Y. Yee J. Gwak H.-S. (2021). Effects of ephedrine-containing products on weight loss and lipid profiles: a systematic review and meta-analysis of randomized controlled trials. Pharmaceuticals 14:1198. doi: 10.3390/ph14111198, PMID: 34832979 PMC8618781

[ref314] Yu D. Xu J. Li R. Zhao J. Li F. Zhai Y.. (2021). Synergetic effect of rifampin loaded mussel-inspired silver nanoparticles for enhanced antibacterial activity against multidrug-resistant strain of *Mycobacterium Tuberculosis*. ChemistrySelect 6, 10682–10687. doi: 10.1002/slct.202101973, PMID: 39714112

[ref315] Yuan X. Zhang X. Sun L. Wei Y. Wei X. (2019). Cellular toxicity and immunological effects of carbon-based nanomaterials. Part. Fibre Toxicol. 16:18. doi: 10.1186/s12989-019-0299-z, PMID: 30975174 PMC6460856

[ref316] Yusuf A. Almotairy A. R. Z. Henidi H. Alshehri O. Y. Aldughaim M. S. (2023). Nanoparticles as drug delivery systems: a review of the implication of nanoparticles’ physicochemical properties on responses in biological systems. Polymers 15:1596. doi: 10.3390/polym15071596, PMID: 37050210 PMC10096782

[ref317] Zahi M. R. El Hattab M. Liang H. Yuan Q. (2016). Enhancing the antimicrobial activity of d-limonene nanoemulsion with the inclusion of ε-polylysine. Food Chem. 221, 18–23. doi: 10.1016/j.foodchem.2016.10.037, PMID: 27979165

[ref318] Zárate S. G. De la Cruz Claure M. L. Benito-Arenas R. Revuelta J. Santana A. G. Bastida A. (2018). Overcoming aminoglycoside enzymatic resistance: design of novel antibiotics and inhibitors. Molecules 23:284. doi: 10.3390/molecules23020284, PMID: 29385736 PMC6017855

[ref319] Zhao G. Usui M. L. Lippman S. I. James G. A. Stewart P. S. Fleckman P.. (2013). Biofilms and inflammation in chronic wounds. Adv. Wound Care 2, 389–399. doi: 10.1089/wound.2012.0381, PMID: 24527355 PMC3763221

[ref320] Zhao X. Yu Z. Ding T. (2020). Quorum-sensing regulation of antimicrobial resistance in Bacteria. Microorganisms 8:425. doi: 10.3390/microorganisms8030425, PMID: 32192182 PMC7143945

[ref321] Zhou L. Zhang Y. Ge Y. Zhu X. Pan J. (2020). Regulatory mechanisms and promising applications of quorum sensing-inhibiting agents in control of bacterial biofilm formation. Front. Microbiol. 11:589640. doi: 10.3389/fmicb.2020.589640, PMID: 33178172 PMC7593269

[ref322] Zhou D. Zhang H. Xue X. Tao Y. Wang S. Ren X.. (2022). Safety evaluation of natural drugs in chronic skeletal disorders: a literature review of clinical trials in the past 20 years. Front. Pharmacol. 12:801287. doi: 10.3389/fphar.2021.801287, PMID: 35095508 PMC8793129

